# Exploring TMPRSS2 Drug Target to Combat Influenza and Coronavirus Infection

**DOI:** 10.1155/sci5/3687892

**Published:** 2025-04-21

**Authors:** Krishnaprasad Baby, Megh Pravin Vithalkar, Somasish Ghosh Dastidar, Chiranjay Mukhopadhyay, Rania Hamdy, Sameh S. M. Soliman, Yogendra Nayak

**Affiliations:** ^1^Department of Pharmacology, Manipal College of Pharmaceutical Sciences, Manipal Academy of Higher Education, Manipal, Karnataka 576104, India; ^2^Centre for Molecular Neurosciences, Kasturba Medical College, Manipal Academy of Higher Education, Manipal, Karnataka 576104, India; ^3^Manipal Institute of Virology, Manipal Academy of Higher Education, Manipal, Karnataka 576104, India; ^4^Department of Microbiology, Kasturba Medical College, Manipal Academy of Higher Education, Manipal, Karnataka 576104, India; ^5^Centre for Emerging and Tropical Diseases, Kasturba Medical College, Manipal Academy of Higher Education, Manipal, Karnataka 576104, India; ^6^Research Institute for Science and Engineering (RISE), University of Sharjah, Sharjah 27272, UAE; ^7^Research Institute for Medical and Health Sciences, University of Sharjah, P.O. Box 27272, Sharjah, UAE; ^8^College of Pharmacy, University of Sharjah, P.O. Box 27272, Sharjah, UAE

**Keywords:** antiviral therapy, coronavirus, COVID-19 treatment, host proteases, influenza virus, protease inhibition, public health strategies, SARS-CoV-2, tTMPRSS2, viral pathogenesis

## Abstract

Respiratory viral infections, including influenza and coronaviruses, present significant health risks worldwide. The recent COVID-19 pandemic highlights the urgent need for novel and effective antiviral agents. The host cell protease, transmembrane serine protease 2 (TMPRSS2), facilitates viral pathogenesis by playing a critical role in viral invasion and disease progression. This protease is coexpressed with the viral receptors of angiotensin-converting enzyme 2 (ACE2) for SARS-CoV-2 in the human respiratory tract and plays a significant role in activating viral proteins and spreading. TMPRSS2 activates the coronavirus spike (S) protein and permits membrane fusion and viral entry by cleaving the virus surface glycoproteins. It also activates the hemagglutinin (HA) protein, an enzyme necessary for the spread of influenza virus. TMPRSS2 inhibitors can reduce viral propagation and morbidity by blocking viral entry into respiratory cells and reducing viral spread, inflammation, and disease severity. This review examines the role of TMPRSS2 in viral replication and pathogenicity. It also offers potential avenues to develop targeted antivirals to inhibit TMPRSS2 function, suggesting a possible focus on targeted antiviral development. Ultimately, the review seeks to contribute to improving public health outcomes related to these viral infections.

## 1. Introduction

Respiratory viral infections, highlighted by the novel influenza and COVID-19, led by many different strains of influenza and coronavirus, have significantly changed life spans and emerged as a prominent public health problem globally [1]. A study of hospitalized people has recently shown that patients who have COVID-19 are at a much higher risk of severe complications than people who come down with the flu. COVID-19 patients have a nearly 19-fold greater risk of developing acute respiratory distress syndrome (ARDS) and more than four times the risk of myocarditis, deep vein thrombosis, pulmonary embolism, and intracranial hemorrhage. When hospitalized, COVID-19 inpatients have a mortality rate of 21.0% compared to only 3.8% for those with influenza. Additionally, the average duration of hospitalization for patients with COVID-19 is almost three times as long. Complications are prevalent in almost every organ system; 70.1% of pneumonia for COVID-19 patients, renal issues 39.6%, and also neurological complications account for 4.1% [2, 3]. In a sizable proportion of cases (17%–53%) of community-acquired severe respiratory disease, predominantly among critically ill patients, infections with respiratory viruses were confirmed; they can induce severe symptoms, like hypoxemic respiratory failure that requires critical care [4].

Influenza A virus (IAV) infections are particularly onerous, frequently resulting in subsequent infections, which increase mortality and morbidity rates [5]. Severe respiratory infections may bring about serious systemic consequences, particularly acute neurologic disorders such as encephalitis and Guillain–Barré syndrome [6]. The advent of severe acute respiratory syndrome coronavirus 2 (SARS-CoV-2) has highlighted the risky nature of coronavirus infections, resulting in common clinical signs with influenza, confounding differential diagnoses amid epidemics [7]. Viral coinfections are widespread, particularly in children with lower respiratory tract infections, and the interactions of diverse viruses can significantly impact disease severity. Cytokine imbalance and immune response changes are two factors linked to the mortality of these diseases [8].

Recent research highlights specific proteins, such as EPAC2 and Galectin-3 binding protein (Gal-3BP), as possible therapeutic avenues for controlling viral replication and inflammation. Elevated Gal-3BP levels are severe COVID-19 cases, contributing to the cytokine storm syndrome by increasing inflammatory cytokines such as IL-1, IL-6, and TNF-α from immune cell populations [5].

Inhibiting Gal-3 can reduce the cytokine release and COVID-19 symptoms, potentially decreasing transforming growth factor-β (TGF-β)-mediated lung fibrosis. Furthermore, a region in the SARS-CoV-2 spike protein mimics human Gal-3, suggesting that targeting it may hinder viral entry and help regulate the immune response [9, 10]. Exchange proteins activated by cAMP (EPAC) play key roles in various disorders, especially infectious diseases. EPAC2 is a vital therapy target for respiratory viral infections, such as respiratory syncytial virus (RSV), as it regulates RSV replication and virus-induced host responses; therefore, blocking EPAC2 reduces RSV proliferation and inflammation, which may lower morbidity and mortality. EPAC2 inhibition can also inhibit the replication of other viruses that affect the respiratory system, such as human metapneumovirus (hMPV) and adenovirus, suggesting its broad therapeutic potential [11, 12]. COVID-19 and influenza are currently being treated with various medications, each with its mechanisms and restrictions. Convalescent plasma therapy (CPT) may lower fatality for COVID-19 patients by giving a form of passive immunity. However, additional trials are required to prove its efficacy [13]. Antiviral medications such as remdesivir RNA-dependent RNA polymerase (RdRp) inhibitor and neuraminidase inhibitors such as oseltamivir, peramivir, and zanamivir are used for influenza, while their effectiveness varies; therapies like hydroxychloroquine (HCQ) have demonstrated conflicting results [14, 15].

HCQ has been studied extensively for its potential in treating COVID-19, yet findings remain inconsistent. A pooled analysis of 11 randomized controlled trials involving 1560 participants with mild COVID-19 revealed no significant benefits in viral load reduction or hospitalization rates compared to control groups [16]. Similarly, another study with 33 participants showed no significant difference in the time to negative viral PCR between HCQ and standard care groups [16]. In severe cases, a phase III trial indicated that HCQ treatment correlated with worse clinical outcomes, including higher rates of invasive mechanical ventilation and renal dysfunction [17]. Another multicenter trial with 479 patients found no significant improvement in clinical status or mortality rates at 14 and 28 days [18].

Additionally, evidence regarding viral clearance with HCQ has been inconclusive, with some studies finding no significant differences in viral clearance over specified periods [19]. Another study with 128 patients found no significant differences in SARS-CoV-2 clearance between HCQ and placebo groups [20]. Safety concerns have also been raised, notably an increased incidence of adverse events and cardiac toxicity among HCQ recipients [21, 22]. The conflicting findings underscore the urgent need for more rigorous and larger-scale studies to evaluate the efficacy and safety of HCQ accurately [19]. The lack of evidence supporting its prophylactic use further emphasizes the necessity for high-quality research to establish definitive conclusions regarding HCQ in COVID-19 treatment.

Immunomodulatory treatments, particularly corticosteroids, contribute to managing severe cases by regulating immune responses [23]. Experimental cell-based therapeutics using mesenchymal stem cells might minimize lung harm from respiratory viruses. Additionally, traditional Chinese medicine, notably Lianhua Qingwen, has demonstrated benefits when combined with conventional therapy for COVID-19 [24]. These current therapeutic strategies for respiratory viral infections are listed in Table 1.

The need for new antiviral medicines is critical, as current alternatives are limited, notably for RSV and other noninfluenza viruses affecting vulnerable populations, such as the elderly, resulting in serious complications such as pneumonia and hospitalization. This emphasizes the significance of comprehensive preventative measures to protect these risk groups [40]. As a result, an in-depth knowledge of viral coinfections, regulatory mechanisms, and epidemiological patterns is required for designing successful strategies to reduce the burden of viral infections of the respiratory tract on public health. Numerous research studies on the impact of coinfections, particularly in children, show significant differences in hospitalization rates, intensive care unit (ICU) admissions, mechanical ventilation needs, or mortality compared to single viral infections [41].

However, certain coinfections, such as those involving RSV and hMPV, are linked to higher ICU admission rates and longer hospital stays. For example, a comprehensive analysis revealed an odds ratio of 7.2 for ICU admission in RSV-hMPV coinfections, implying that specific viral pairings may worsen disease severity compared to others [42]. In contrast, in instances of COVID-19, the existence of coinfections with additional respiratory viruses was relatively rare. It did not seem to worsen disease severity significantly. A U.K. study found that only 2% of hospitalized COVID-19 patients had viral coinfections without a notable increase in adverse outcomes [43, 44]. Bacterial coinfections were typical but did not affect hospitalization length or fatality rates [45]. Though coinfections are possibly more frequently occurring in severe respiratory infections, their effect on the extent of the illness differs by the pathogen, demanding additional research to understand these complicated relationships and their clinical consequences.

TMPRSS2 is a type II transmembrane serine protease involved in the pathological process of numerous viral diseases, including coronaviruses like SARS-CoV-2, SARS-CoV, and MERS-CoV. TMPRSS2 is primarily expressed in the epithelial cells that line the pulmonary and digestive systems, where it cleaves viral proteins, enabling the fusion of membranes and increasing viral infectivity [46]. It is essential in activating viral S proteins in primary human bronchial cells and IAV and influenza B virus (IBV) in primary human type II pneumocytes that cause membrane fusion [47]. TMPRSS2, co-expressed with ACE2 in type II pneumocytes, plays an essential role in viral activation by cleaving the spike (S) protein in SARS-CoV and SARS-CoV-2 and hemagglutinin (HA) in influenza viruses, facilitating viral entry into host cells and promoting infectivity and spread in the lungs [47]. TMPRSS2 and TMPRSS4 promote the trypsin-independent propagation of influenza virus in Caco-2 cells, indicating their function in viral activation in naturally receptive cells [47]. Research suggests that the lack of TMPRSS2 dramatically reduces the pathogenicity of coronaviruses in animal models, indicating its critical involvement in viral transmission and inflammatory responses. TMPRSS2 expression levels also alter the SARS-CoV-2 entry mechanism; decreased levels lead to endosomal entrance via cathepsin L (CTSB/L), whereas elevated levels enable direct membrane fusion [48]. Additionally, TMPRSS2 regulates immunological responses during infections, and research indicates that TMPRSS2-deficient animals had altered cytokine profiles, reduced inflammatory responses, less weight loss, reduced pulmonary viral load, and less lung injury than wild-type mice, indicating the importance of TMPRSS2 in activating S protein for viral entry and propagation [46].

Inhibitors such as camostat and nafamostat have shown guarantee in hindering TMPRSS2 activity, possibly preventing virus entry and reproduction, offering an intriguing treatment option for addressing influenza and coronavirus infections to prevent viral entry while concurrently modulating the inflammatory response to reduce disease extent [48]. Addressing TMPRSS2 is a viable technique for developing antiviral medications for influenza and coronaviruses, emphasizing its role in viral pathogenesis and therapy. Another study discovered that TMPRSS2 is critical to transmitting the H1N1 influenza virus [49]. Researchers found that knocking down TMPRSS2 in mice lowered viral spread, guarding against weight loss, mortality, and lung damage [50].

Because of the crucial function of TMPRSS2 in virus entry and disease progression, and by concentrating on possible inhibitors, this article attempts to synthesize existing understanding of TMPRSS2. It provides insights into innovative treatment tactics for influenza and coronaviruses. This can effectively prevent viral entrance and replication, laying the groundwork for developing novel antiviral medications, decreasing the extent of infections and transmission rates, and dealing with pressing public health concerns. We also advocate for more extensive clinical trials and research to evaluate the safety and effectiveness of new therapeutic agents targeting this protease, which might lead to viable therapies for respiratory viruses.

## 2. TMPRSS2 Gene and Protein Structure

**Figure 1:**
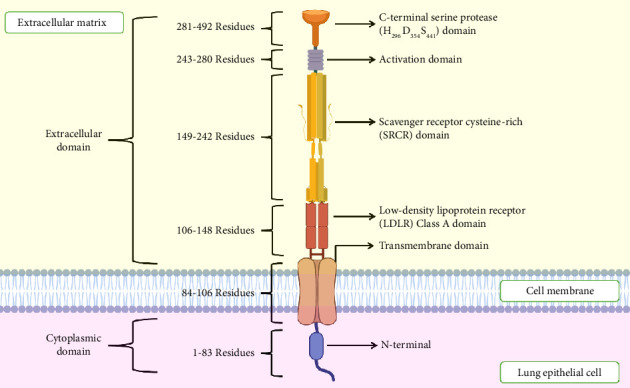
A detailed structure of TMPRSS2 protein and its domains. This is an original figure created by the authors using BioRender (under an academic license, MAHE, Manipal, India) and PowerPoint.

TMPRSS2 gene, identified on chromosome 21q22.3, is highly conserved throughout species. It exists in two isoforms, both autocatalytically activated through their dormant zymogen states. This gene is expressed in several organs, with notably substantial expression in the small intestine and comparably in low levels in different tissues [51, 52]. TMPRSS2 resides mainly on the apical surface of luminal epithelial cells in the prostate gland. It gets released into semen as a component of the organelle-like vesicles called prostasomes [53]. The TMPRSS2 protein contains 492 amino acids and includes a short cytoplasmic tail, a transmembrane domain, a stem region with CUB and LDL receptor-like domains, and a carboxy-terminal serine protease (amino acids 255–492) domain, which is required for the proteolytic activity, likely cleaving at arginine or lysine residues; the scavenger receptor cysteine-rich (SRCR) domain (amino acids 149–242) for binding to other cell surface or extracellular molecules; the low-density lipoprotein receptor class A (LDLRA) domain (amino acids 113–148) for calcium binding; and the transmembrane domain (predicted at amino acids 84–106) for anchoring the protein to the cell membrane as shown in Figure 1. TMPRSS2 is a target for prospective therapeutic interventions against COVID-19 as it is an essential part of several biological processes, including viral entry by priming the S protein of viruses like SARS-CoV and SARS-CoV-2, crucial for their infectivity [52].

TMPRSS2 is commonly linked to carcinogenesis by its involvement in gene fusions with the ERG gene; TMPRSS2–ERG fusion is the most prevalent chromosomal abnormality in prostate carcinoma [51]. Furthermore, androgens influence the expression of TMPRSS2. Through the activation of proteolytic cascades involving elements of the tumor microenvironment, it is linked to the spread of prostate cancer [54]. Overall, TMPRSS2 emerges as a complex gene with important implications for viral pathogenesis and the biology of cancer, illustrating its role as a serine protease that fosters critical biological functions, as well as an intriguing target for COVID-19 therapy and a critical biomarker for cancers of the prostate, its diagnosis, and prognosis.

### 2.1. Coexpression of TMPRSS2 With Viral Receptors in the Human Respiratory Tract

TMPRSS2 and HAT are co-expressed with 2,6-linked sialic acids, a key receptor driver of human influenza viruses throughout the human respiratory tract, which encourage viral transmission [55]. The expression levels of TMPRSS2 and ACE2 were linked to the prognoses of COVID-19 patients. For example, mutations in the TMPRSS2 gene have been correlated with varied susceptibilities to SARS-CoV-2 infection, illustrating that variations in the genome can alter the expression of these receptors and, as a result, the clinical manifestations of the disease [56]. The coexpression of TMPRSS2 with viral receptors, which ACE2 in epithelial cells across the respiratory tract (excluding the vocal folds, epiglottis, and trachea) facilitates SARS-CoV-2 entry [55].

Elevated levels in nasal passages, airways, and alveoli have been linked to respiratory symptoms and virus transmission dynamics [55, 57]. Coexpression in the digestive system offers a possible entrance route for SARS-CoV-2 through the gastrointestinal tract, demonstrating the virus's complicated infection patterns across many organs [58]. The levels of TMPRSS2 and ACE2, which are primarily expressed in bronchial transient secretory cells, are associated with the clinical outcomes of COVID-19 patients. This link is significant as it indicates that addressing TMPRSS2 might successfully disrupt the early phases of viral infection. Clinical investigations have found a link between TMPRSS2 expression levels and COVID-19 results, implying that TMPRSS2 polymorphisms can alter the propensity to diseases and their severity. This diversity, driven by demographic characteristics such as age and gender, underscores the value of understanding the role of this protease in virus entry and the larger context of pathogenicity [59].

In human pulmonary epithelial cells, TMPRSS2 is co-expressed along CD13, the receptor for human coronavirus 229E (HCoV-229E), indicating that TMPRSS2 may trigger HCoV-229E in infected humans [60]. Given its critical involvement in viral entry, TMPRSS2 is a prospective target for antiviral treatment. The inhibitors of TMPRSS2 function may impede the activation of S proteins, thus hindering viral entry. This treatment method may be beneficial for treating illnesses caused by influenza and coronaviruses [48, 61]. As a result, comprehending how TMPRSS2 interacts with viral receptors can aid in advancing these research techniques, making them critical for contending respiratory infections. Understanding the role of this protease in viral entry and its consequences for the extent of the disease provides a solid platform for investigating inhibition techniques. This field of research is highly significant because it has the potential to significantly enhance outcomes from therapy for influenza and coronavirus infections by tailored inhibition of TMPRSS2.

### 2.2. TMPRSS2 Expression in Relation to Virus Type, Tissues, and Ethnicity

The process of viral entry into the host cells and the development of viral infections via membrane fusion by TMPRSS2 emphasizes the relevance of this protease in viral entry [62]. The expression of protease is colocalized with viral receptors in the respiratory tract, enhancing the infectivity of both coronaviruses SARS-CoV and SARS-CoV-2 and influenza viruses [55]. Additionally, research conducted in mouse models shows that the lack of TMPRSS2 considerably lowers lung pathology and viral spread following SARS-CoV and MERS-CoV infections, indicating that it plays an important role in both viral pathogenesis and immunopathology. It decreases viral spread in mouse models, demonstrating its effect on disease severity and immunopathology [46]. Studies have demonstrated that TMPRSS2 enhances the spread of viruses through localized infections and facilitates syncytium development, enabling effective cell-to-cell dissemination while escaping immune responses [63]. Given its critical roles in viral activation and propagation, blocking TMPRSS2 might significantly limit viral infectivity and dissemination, making it an appealing target for therapeutic therapies.

TMPRSS2 expression varies widely between populations, which influences COVID-19 susceptibility and severity. Genetic investigations show that African populations have low TMPRSS2 expression levels, which might decrease their vulnerability to infection [46, 64]. European and American populations exhibit elevated expression levels, indicating a greater risk. Specific single-nucleotide polymorphisms (SNPs), such as rs12329760, vary in frequency between ethnic communities; for example, this mutation is more common in Asian people and can provide some protection from severe COVID-19 symptoms [65, 66]. Furthermore, TMPRSS2 expression is maximal in some organs, including the prostate, stomach, pancreas, lungs, small intestine, and salivary gland. It appears to grow with time, especially in men, and further influences the consequences of diseases [57, 67].

It has also been implicated in other respiratory viruses, such as influenza, wherein this protease facilitates the spread of these viruses by enabling them to bypass the need for trypsin-like proteases, which are typically required for their activation [61]. The clinical relevance is reinforced by findings indicating that symptomatic COVID-19 patients exhibit higher TMPRSS2 expression levels than asymptomatic individuals, suggesting that targeting this protease could be particularly beneficial in treatment strategies aimed at symptomatic patients. TMPRSS2 expression is crucial in recruiting various immune cells, potentially impacting both the immune response and disease progression [68]. Higher TMPRSS2 expression in males, African Americans, and diabetics may lead to increased susceptibility and worse COVID-19 outcomes. Inhaled corticosteroids are linked to lower TMPRSS2 expression, potentially offering protection against severe disease [57, 69]. Genetic variations in TMPRSS2 alter COVID-19 patient's vulnerability and disease intensity [56]. A continued study of the processes and expression characteristics of TMPRSS2 is critical for developing antiviral treatments and vaccines against viral infections of the respiratory tract.

### 2.3. TMPRSS2 in Activating Viral Proteins and Mechanism of Action as a Serine Protease

**Figure 2:**
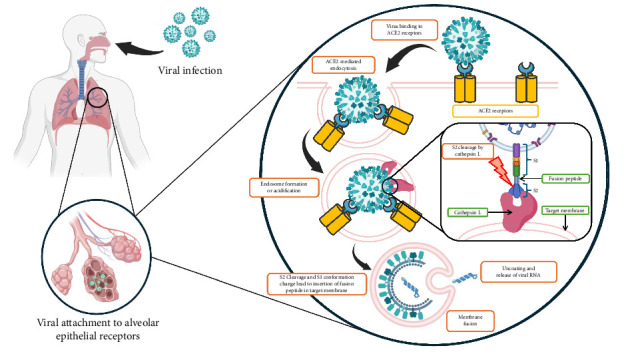
Mechanism of viral entry in the absence of TMPRSS2. This is an original figure created by the authors using BioRender (under an academic license, MAHE, Manipal, India) and PowerPoint.

TMPRSS2 is a key serine protease that splits the S protein of coronaviruses like SARS-CoV and MERS-CoV, enhancing the viral entry by activating proteins important for membrane fusion, specifically through the S protein of coronaviruses. This protease cleaves the host cell receptor ACE2, increasing viral uptake and boosting pathogenicity in respiratory and gastrointestinal organs where TMPRSS2 is heavily expressed [70]. SARS-CoV-2's S protein initially links to the host ACE2 receptors. In the setting of viral entry, TMPRSS2 collaborates alongside other proteases, particularly cathepsins. TMPRSS2 activates the S protein without cathepsins, suggesting additional viral entry routes [60]. Cathepsins are cysteine proteases that cleave viral glycoproteins, hence facilitating viral entrance. TMPRSS2 and cathepsins are important in viral protein activation; however, targeting TMPRSS2 may provide specific benefits. TMPRSS2 triggers the S protein on the cell surface, whereas endosome cathepsins suggest alternative entrance paths [71]. TMPRSS2 is, for example, predominantly expressed on the cell exterior of epithelial cells, which renders it more accessible to inhibitors than cathepsins, which are frequently intracellular. Furthermore, blocking TMPRSS2 may directly impede the first processes of viral entry, perhaps lowering viral load more effectively than targeting subsequent procedures controlled by cathepsins. TMPRSS2 or cathepsin inhibitions hinder viral entrance; however, combination inhibition of both considerably increases antiviral effects [72]. The concept is pictured in the diagram in Figure 2 below. It shows both the alternative entry pathway and how cathepsins are involved with spike protein activation.

In influenza viruses, TMPRSS2 activates the HA protein that is required for viral infectivity and replication; knockout (KO) models indicate poor HA cleavage and diminished viral dissemination when TMPRSS2 is lacking [47]. The expression of TMPRSS2 across multiple tissues, particularly in the intestinal and respiratory tracts, coincides with common locations of viral infection, indicating its function in viral transmission and pathogenesis [73]. Given its substantial role in viral protein activation, including hormonal modulation, and its association with other proteases that may alter cell sensitivity to viral infections, TMPRSS2 is a viable therapeutic target. Inhibitors of TMPRSS2 have demonstrated potential for preventing viral activation and dissemination, emphasizing the requirement for additional studies to develop efficient antiviral methods toward respiratory tract viruses like COVID-19 and influenza.

### 2.4. Cleavage of Viral Surface Glycoproteins by TMPRSS2

TMPRSS2 is the primary protease accountable for the cleavage and activation of HA in IAV and IBV in human airway cells, which is required for viral infectivity and dissemination. This activation is required for viral entrance to host cells, demonstrating the protease's critical function in the viral lifecycle. TMPRSS2 cleaves the freshly generated HA protein of IAVs and IBVs. Other proteases, such as HAT, break HA at the cell surface, suggesting distinct subcellular localizations and methods of action, and are crucial for viral infectiousness and propagation, suggesting that inhibition of TMPRSS2 can greatly diminish viral replication [74]. In the case of SARS-CoV-2, TMPRSS2 cleaves the S protein at the S2′ location, which is positioned right ahead of the hydrophobic fusion peptide, revealing the fusion peptide, allowing membrane fusion and boosting viral penetration [75]. This protease cleaves the ACE2 receptor, which increases viral infection capacity [70]. The nonprotease domains of TMPRSS2, including its cytosolic tail and SRCR domain, play roles in regulating SARS-CoV-2 entry pathways, adding complexity to its function [76]. Research has demonstrated that novel inhibitors targeting TMPRSS2 can effectively stop the proliferation of influenza viruses and SARS-CoV-2, indicating a possible treatment route [77]. According to investigations, TMPRSS2 cleavage activity is distinct yet complementary to other proteases, such as furin, which break down viral glycoproteins. Combining TMPRSS2 inhibitors with furin inhibitors improved antiviral efficacy toward SARS-CoV-2. Phytopharmaceuticals inhibiting TMPRSS2 and furin demonstrate potential in treating COVID-19 by preventing SARS-CoV-2 entrance and replication, lowering fatality rates [78]. TMPRSS2 and furin are crucial for activating the viral spike protein. Hence, they are key targets for antiviral treatments. Arbidol analogs can inhibit several targets, including ACE2, which increases its efficacy. A combination therapy that suppresses TMPRSS2 and furin has shown a substantial decrease in viral infection, with particular combinations attaining up to a 95% drop in lung cells. Modified serpin inhibitors have also improved effectiveness in inhibiting viral entry [79].

Virtual screenings have revealed phytochemicals, such as limonin and gedunin, as possible inhibitors for these proteases [80]. Ongoing clinical trials of arbidol and research into synthetic inhibitors like decanoyl-RVKR-chloromethyl ketone (CMK) and naphthofluorescein indicate an excellent potential for a combination approach against COVID-19 [81, 82]. As a result, studying TMPRSS2 methods of action and subcellular location provides insights into designing effective antiviral therapies, establishing it as a key target for therapeutic intervention toward viral infections.

### 2.5. Activation of Coronavirus S Protein and Influenza HA Protein

HA and S proteins are extensively glycosylated, making them susceptible to carbohydrate-binding agents like lectins. The lectin Flt3 receptor-interacting lectin (FRIL) from Lablab beans can neutralize influenza viruses and SARS-CoV-2 by binding to complex-type N-glycans on their envelopes and blocking viral entrance into host cells [83]. Coronavirus hemagglutinin-esterase (HE) and S proteins are essential for effective viral attachment and entry [84]. Coronavirus S proteins, such as SARS-CoV-2, are trimeric glycoproteins that attach to host cell receptors and facilitate membrane fusion [85]. TMPRSS2 and TMPRSS11A serve as essential type II transmembrane serine proteases, which activate the HA protein of IAVs and S protein of coronaviruses, providing viral entry into host cells, fostering viral spread, and decreasing the efficacy of the humoral immune response by decreasing antibody recognition [86]. S protein binds to the ACE2 receptor on host cells, mainly via the S protein receptor-binding domain (RBD) [85]. Consequently, when bound, it causes a conformational shift that exposes fusion peptides required for membrane fusion.

**Figure 3:**
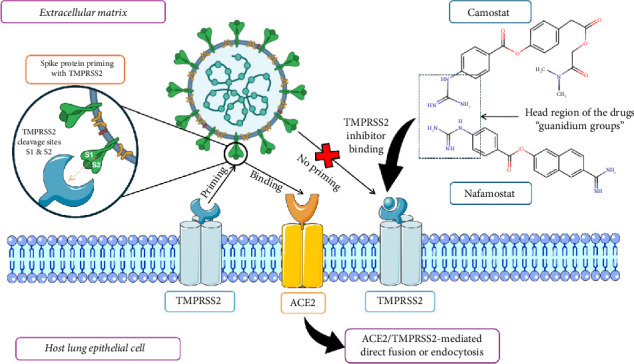
Mechanism of viral entry into the host cell and the therapeutic activity of TMPRSS2 inhibitors. This is an original figure created by the authors using BioRender (under an academic license, MAHE, Manipal, India) and PowerPoint.

S protein is a precursor protein (S0) that needs proteolytic cleavage. This cleavage happens at the S1/S2 junction, exposing the fusion peptide in the S2 subunit, as depicted in Figure 3. A furin cleavage site in SARS-CoV-2 increases its ability to infect by permitting more efficient processing by host proteases, like furin, in various tissues. Following receptor engagement and cleavage, the S protein switches from a prefusion to a postfusion setup. This transition is marked by the S2 subunit bending back, bringing the fusion peptide nearer to the host cell membrane and promoting the fusion of the membrane. Cryo-electron microscopy-based structural investigations have revealed these conformational changes, exposing the S protein's dynamic nature upon entrance. This is critical for entering viruses into host cells, which is required for comprehending viral pathogenesis and transmission. TMPRSS2 remarkably improves the ability of SARS-CoV infection by splitting its S protein, which lowers the efficiency of the immune response by decreasing antibody recognition [75, 87–91].

HA protein of influenza viruses has an analogous function in viral entrance but via different processes. While TMPRSS11A may stimulate HA, the cellular serine protease inhibitor HAI-1 does not block it, suggesting a distinct sensitivity across these proteases [86]. HA protein of influenza viruses has a similar role in viral entrance but operates through different methods. TMPRSS11A can activate HA, but the cellular serine protease inhibitor HAI-1 does not block it, showing a distinct sensitivity among these proteases [83]. Viral invasion begins when HA attaches to sialic acid residues on the outermost layer of host cells. This binding is critical to defining host tropism. The type of sialic acid linkage (α2, 3 or α2, 6) found in target cells affects this [92, 93]. HA, like S protein, requires proteolytic cleavage to be initiated. This cleavage normally happens at particular locations within the HA molecule, changing it from an inactive precursor (HA0) to its active forms (HA1 and HA2) via host proteases, such as trypsin or plasmin, found in respiratory epithelial cells. When HA binds to and cleaves a receptor, it experiences considerable conformational changes that expose its fusion peptide. These modifications are necessary for the membranes of cells and viruses to fuse. Structural investigations have revealed that HA moves from a prefusion to a postfusion state, comparable to coronaviruses. This transformation is also necessary to facilitate viral entrance into host cells [94, 95].

### 2.6. Viral Entry via Membrane Fusion Facilitated by TMPRSS2

TMPRSS2 enhances viral entrance efficiency by cleaving S protein, allowing the virus to fuse to the host cell's outer membrane. This increases viral infectiousness greatly as compared with receptor-mediated endocytosis. TMPRSS2 interacts with other proteases, such as ADAM17, for ACE2 processing, but only TMPRSS2 cleavage leads to increased viral entrance. This improves viral infectiousness by encouraging direct fusion with the host cell membrane, therefore improving the efficiency of viral entry compared to receptor-mediated endocytosis [70, 73]. The co-expression of TMPRSS2 and ACE2 in target cells correlates with the sites of SARS-CoV-2 infection, highlighting TMPRSS2 role in viral tropism and pathogenicity [73]. TMPRSS2 cleaves the SARS-CoV-2 S protein at two important sites: the S1/S2 site, which contains the furin cleavage site, and the S2′ site. Furin mediates the S1/S2 cleavage during viral assembly. TMPRSS2 also cleaves at the S2′ location after the S protein attaches to its receptor, ACE2. This cleavage is necessary for modifications to conformation in S protein, which facilitates membrane fusion and subsequent entry of viruses into the host cells. When TMPRSS2 binds to ACE2, it cleaves the S protein at the S2′ location, causing conformational changes that result in the fusion of membranes between the virus and host cells. This mechanism is necessary for RNA viruses to enter into the host cell cytoplasm. This cleavage is required for conformational modifications of the spike protein, facilitating membrane fusion and subsequent viral entry into target cells. Upon binding to ACE2, TMPRSS2 cleaves the S protein at the S2′ location, causing conformation modifications that result in membrane fusion among the viral and host cells. This mechanism is required for viral RNA to enter the host cell cytoplasm [75, 85, 96].

SARS-CoV-2's reliance on TMPRSS2 emphasizes a shared mechanism across coronaviruses, similar to SARS-CoV-1 and MERS-CoV, which utilizes host proteases to activate S proteins. Inhibition of TMPRSS2 is a viable treatment method for reducing infection rates since it directly affects viral entry processes. Furthermore, TMPRSS2 nonprotease domains may modulate viral entry pathways, and its effect on cytokine release has bigger consequences for immune response regulation. Recognizing the biochemical mechanisms and structural interactions of TMPRSS2 with the S protein highlights its possibility as a therapeutic target against COVID-19 and other viruses that employ similar entry pathways.

## 3. TMPRSS2 Activity-Dependent Viral Entry

**Figure 4:**
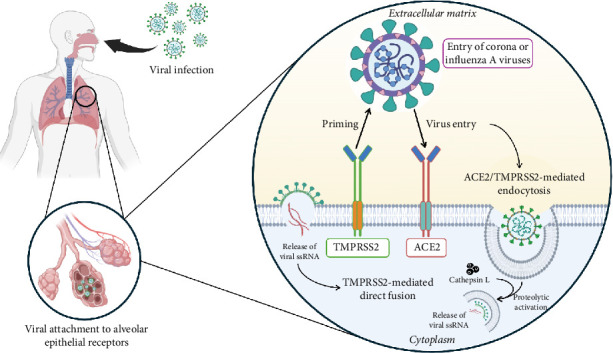
Mechanism of coronavirus entry into the host cell via ACE/TMPRSS2-mediated direct fusion or endocytosis. This is an original figure created by the authors using BioRender (under an academic license, MAHE, Manipal, India) and PowerPoint.

TMPRSS2 in respiratory epithelial cells cleaves the viral S protein, allowing SARS-CoV-2 to enter the host cell by membrane fusion. TMPRSS2 competes against other proteases, such as ADAM17, for processing ACE2, but only TMPRSS2 cleavage improves viral entry [70]. Without TMPRSS2, SARS-CoV-2 may get into cells through endocytosis and cathepsin L activation, but this route is less effective. The presence or absence of TMPRSS2 determines whether SARS-CoV-2 enters cells by direct fusion of membranes or endocytosis [64]. The presence or absence of TMPRSS2 determines whether SARS-CoV-2 enters cells directly by membrane fusion or endocytosis [60] (Figure 4).

TMPRSS2 has three structural components: an N-terminal cytosolic domain, a single transmembrane domain, and a serine protease component that is accountable for its enzyme activity. TMPRSS2 contains a trypsin-like serine protease motif that breaks down particular peptide bonds in target proteins. The cleavage at certain locations activates the protease, allowing it to communicate with substrates such as viral S proteins [97]. TMPRSS2 is required to activate the SARS-CoV-2 S protein, which mediates viral entrance into host cells via membrane fusion. SARS-CoV-2 S protein changes shape when it binds to host cells' ACE2 receptor. This interaction is required for TMPRSS2 to cleave S protein at certain sites (e.g., S1/S2 cleavage site and S2′ site), which are important for the fusion of the membrane [75, 96].

TMPRSS2 plays a critical role in viral infection by cleaving S protein at both the S1/S2 boundary and the S2′ site, exposing the fusion peptide. Following its cleavage, the S protein undergoes a major conformational change, bringing the viral and host cell membranes close to facilitate their union [85, 98]. This fusion process is necessary because it permits the viral RNA to enter the host cell cytoplasm and commence infection. The molecular pathways involved in membrane fusion include several important steps: First, the S protein attaches to the ACE2 receptor on host cells; then, TMPRSS2 cleaves the S protein, activating it for fusion; and last, the S protein undergoes a conformational shift, exposing its fusion peptide. Membrane hemifusion develops as the cellular and viral membranes fuse at a lipid bilayer level, eventually creating a pore that allows viral RNA access into the host cell [75, 99, 100].

This protease specifically cleaves at sites on S protein, facilitating efficient viral entry compared to alternative pathways such as endocytosis, which is less effective in the absence of TMPRSS2. The expression of TMPRSS2 in respiratory epithelial cells correlates with an increased susceptibility to SARS-CoV-2 infection, highlighting its importance as a therapeutic target [94]. Inhibitors of TMPRSS2 have shown potential in preventing viral entry, especially via the membrane fusion pathway prevalent in cells expressing this protease [48, 95]. The article also includes ongoing investigations into comprehending TMPRSS2 interactions with various viral protein types and developing small-molecule inhibitors to improve antiviral methods. Introducing SARS-CoV-2 mutations may affect TMPRSS2-mediated entrance, prompting additional research into their susceptibility to current therapeutic strategies.

### 3.1. TMPRSS2-Dependent Propagation of Viruses

Investigations have revealed that the KO of TMPRSS2 dramatically lowers viral proliferation in cell lines and that TMPRSS2 KO animals demonstrate a decreased HA cleavage and diminished infectivity of low-pathogenic IAVs, highlighting its crucial role [48]. TMPRSS2 is also required to activate S protein in coronaviruses like SARS-CoV, SARS-CoV-2, and MERS-CoV. This promotes their entrance into host cells. Enhanced expression of TMPRSS2 in human airway epithelial cells correlates with greater susceptibility to SARS-CoV-2 infection, promoting both viral isolation and dissemination [70].

Similarly, TMPRSS2 triggers HCoV-229E's S protein, a coronavirus linked to the common cold. This activation enables the virus to enter host cells without cathepsin L, a protease implicated in viral entrance. The co-expression of TMPRSS2 with the HCoV-229E receptor CD13 in human airway epithelial cells indicates that TMPRSS2 plays an important role in the infection process caused by HCoV-229E [60, 101]. Targeting TMPRSS2 has substantial pharmacological potential; inhibitors have been reported to impede the growth of influenza viruses and potentially other respiratory viruses. For example, tiny compounds that stabilize G-quadruplex structures in the TMPRSS2 gene promoter can downregulate its production, suppressing influenza virus propagation [60, 102].

### 3.2. Role of TMPRSS2 in Influenza Infection

The activation of influenza virus can also be regulated by genetic and environmental factors, making its role in influenza infection complex. This protease is highly expressed in the human respiratory tract epithelium. Highly expressed proteases can be traced in the small airway and nasal epithelium, where they share a colocalization pattern with sialic acid receptors, the main binding sites of influenza viruses. In contrast, masticatory mucosa has lower expression of this enzyme [103–105]. This enzyme is the main target cell for influenza virus infection. The proximity of the HA and NA proteins facilitates the cleavage of the virus and the subsequent activation of the virus after it enters the host to promote the spread of viral infection [106, 107].

**Figure 5:**
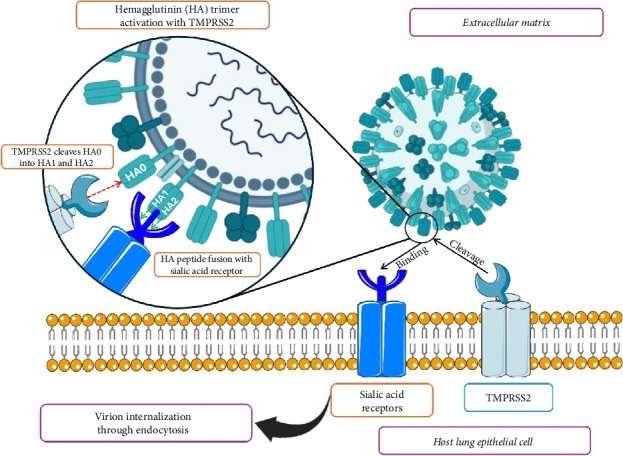
Mechanism of influenza virus entry into the host cell via sialic acid receptor and TMPRSS2-mediated endocytosis. This is an original figure created by the authors using BioRender (under an academic license, MAHE, Manipal, India) and PowerPoint.

As shown in Figure 5, one of the key proteases that cleave and activate viral HA protein is TMPRSS2, a critical component of the virus fusion with the host cell membrane and for initiating infection [47]. This enables the entry and spread of influenza viruses within the host cells. This is further confirmed by the observation that TMPRSS2 colocalizes with HA protein in lung-derived cell lines. Studies in human airway cells and animal models (mice and pigs) have shown that TMPRSS2 was crucial for HA cleavage. KO of TMPRSS2 in human bronchial epithelial cells and type II alveolar epithelial cells markedly reduces the activation and dissemination of IAV [108]. In particular, TMPRSS2 requires a monobasic cleavage site, that is, a single arginine residue, to cleave HA. This cleavage is necessary for the HA protein to transition from its precursor form (HA0) to its active forms, HA1 and HA2, for the virus to fuse to membranes of host cells and begin infection [47, 109, 110]. This process is required for viral entry into host cells, such as H1N1 and H7N9 IAV subtypes. It is crucial for viral membrane fusion and RNA entry into the cell, indicating that there is a differential dependence on TMPRSS2 between different influenza subtypes and allowing for initiating infection [111]. In particular, it is the activation function that plays a fundamental role in the propagation and pathogenesis of some IAV subtypes, such as H1N1 and H7N9, within the host organism, with diminished viral replication and pathogenicity observed in TMPRSS2 KO mice. H1 and H7 subtypes, however, have lower replication and pathogenicity due to HA cleavage deficiency.

Genetic factors such as polymorphisms that moderate the expression of TMPRSS2 have demonstrated predictive value in the susceptibility and severity of influenza infections. TMPRSS2 expression in the respiratory tract is regulated by post-translational modifications of TMPRSS2, including zymogen activation by autocatalysis in the intracellular environment. Moreover, N-glycosylation at a conserved site is important for the correct folding of the protein, its intracellular trafficking, and its expression on the cell surface [97]. Polymorphisms of the TMPRSS2 gene have been shown to affect susceptibility to and severity of influenza infections. Another example would be the SNP, rs2070788, which has been associated with increased TMPRSS2 expression levels, suggesting the contribution of TMPRSS2 to severe influenza A (H1N1) pdm09 and A (H7N9) infections. A second SNP, rs383510, which lies in a regulatory region, similarly influences TMPRSS2 expression and is associated with susceptibility to these influenza strains [112]. In the context of COVID-19, the rs12329760 polymorphism (Val ⟶ Met substitution) has been evaluated. However, it could also be relevant in the case of influenza virus infection [113, 114].

Additionally, rs12329760, a missense mutation located in exon 6/7 of the TMPRSS2 gene, is linked to regional-specific differential distribution, as well as being relatively more common in Asian populations compared to European ones. These genetic alterations further mediate the regulation of TMPRSS2 expression [115]. TMPRSS2 has transcriptional regulation under the influence of cytokines and androgens. It is an androgen-regulated gene expressed in higher doses in men, particularly those older than 70. Androgen deprivation therapies (ADTs) have been shown to downregulate the enzyme and may provide protective effects against SARS-CoV-2 [66, 115].

Furthermore, Th2 cytokines can decrease TMPRSS2 expression in nasal epithelial cells, especially in patients with chronic rhinosinusitis with nasal polyps (CRSwNP) [116]. Co-expression of the protease with 2,6-linked sialic acids, the primary receptor determinant of human influenza viruses, across the human respiratory tract substantiates its contribution to the spread of the virus in humans [116]. This colocalization also positions viral particles close to the protease necessary for their activation. It allows the transport of HA protein for efficient cleavage immediately following receptor binding and facilitates cleavage and activation of the virus following entry into the host and spread [110].

TMPRSS2 is essential for activating IAV subtypes with monobasic cleavage sites, but not for others. H3N2, for example, can use alternate proteases to cleave HA. Of significance, TMPRSS4 can also cleave and activate H3N2 HA in the absence of TMPRSS2, as shown in KO experiments using mouse models [117–119]. Moreover, while mouse hepsin and prostasin can cleave and activate H3 HA, their human equivalents are not functional, highlighting species-specific differences [117]. Additionally, TMPRSS13 has also been shown to be involved in HA activation, suggesting that the protease used to process H3N2 must be somewhat redundant. This redundancy protects the virus from extinction by the host seeking out and eliminating all of its specific proteases [117]. Such redundancy poses challenges for developing effective antiviral therapies because different species of protease activity may limit the transferability of animal model findings to human disease. However, the fact that TMPRSS2 is not the only protease involved in the facilitation of cell entry and spread of SARS CoV-2 suggests that inhibition of other proteases, including TMPRSS4, may represent a new therapeutic avenue to address mitigation of SARS CoV-2 spread and viral pathogenesis.

By contrast, the IBV needs no TMPRSS2 for replication, further emphasizing the specialized role of the enzyme in IAV. This serine protease is normally expressed in respiratory epithelial cells, including colocalization with sialic acid receptors, decreasing the viral activation [117]. Due to its central role, TMPRSS2 is a potential target for antiviral therapy, and inhibitors have been shown to lower the spread of the virus [120]. Because TMPRSS2 is central to IAV activation, we present it as a potential target for antiviral therapies. TMPRSS2 inhibitors have also been effective in blocking viral dissemination, indicating a key role of the enzyme in the pathogenesis of IAVs. In contrast, IBVs can replicate without TMPRSS2, highlighting the unique aspect of IAV that requires TMPRSS2.

## 4. Role of TMPRSS2 in Morbidity Caused by Respiratory Tract Infections

**Figure 6:**
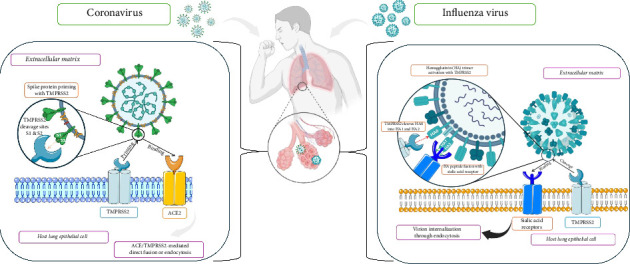
Role of TMPRSS2 in respiratory viral infections caused by influenza and coronaviruses. This is an original figure created by the authors using BioRender (under an academic license, MAHE, Manipal, India) and PowerPoint.

Respiratory tract infections, especially those caused by infectious viruses such as SARS-CoV-2 and influenza, are responsible for substantial mortality and morbidity worldwide. TMPRSS2 is now recognized as a significant element in the infectivity and pathogenesis of many viral infections, making it essential to discover possible treatment strategies [50, 97]. As depicted in Figure 6, TMPRSS2 is essential for the entry of SARS-CoV-2 and influenza virus into host cells, as it facilitates the cleavage of S protein and HA, respectively, a process vital for viral fusion and subsequent entry into the cell [110, 121]. Variations in the genetic makeup of TMPRSS2 could be responsible for the disparities in COVID-19 severity found across different groups of people, suggesting that people carrying specific TMPRSS2 variants may have a higher risk of experiencing severe health consequences [96, 122]. Inhibition of TMPRSS2 has been found to hinder the activation and proliferation of influenza viruses, indicating its potential as a therapeutic target [47].

Inhibitors such as camostat have shown success in lowering SARS-CoV-2 infection in lung cells, indicating that targeting TMPRSS2 could be an attractive option for addressing COVID-19; additionally, when paired alongside other protease inhibitors such as furin inhibitors, these TMPRSS2 inhibitors have shown a combined effect that significantly diminishes viral entry and infection rates [95, 123]. The high expression of TMPRSS2 across multiple organs, especially the lungs, prostate, and gastrointestinal tract, may shed light on its role in the propagation and extent of respiratory infections. More precisely, its expression in bronchial transient secretory cells and type II pneumocytes highlights its relevance in aiding viral entry and replication in the respiratory tract [124, 125]. As a result, the critical involvement of TMPRSS2 in the infectivity and pathophysiology of viral respiratory infections such as SARS-CoV-2 and influenza makes it an attractive target for therapeutic interventions to reduce death and disability caused by these respiratory viruses [61].

### 4.1. Comparative Analysis of TMPRSS2 Role in Different Coronaviruses

TMPRSS2 is a host cell protease that activates S proteins of numerous coronaviruses, including SARS-CoV, MERS-CoV, SARS-CoV-2, and HCoV-229E. This comparative investigation focuses on TMPRSS2 role in various viruses, demonstrating its major impact on viral infection and pathogenesis. In SARS-CoV and MERS-CoV, TMPRSS2 enhances virus–cell membrane fusion at the cell surface, and studies show that its deletion leads to reduced lung pathology and viral propagation in mice models [126, 127]. For SARS-CoV-2, TMPRSS2 is essential for S protein cleavage, enhancing viral entry through membrane fusion rather than receptor-mediated endocytosis [76]. TMPRSS2 also activates HCoV-229E's S protein, independent of cathepsin L [60]. Given its critical role, TMPRSS2 is a prospective target for antiviral therapy; inhibitors such as camostat have demonstrated efficacy in limiting viral entrance in cell cultures. The findings highlight the necessity for additional research into TMPRSS2 inhibitors to address upcoming and future coronavirus outbreaks.

### 4.2. Current Therapeutic Strategies Targeting TMPRSS2

Current treatment approaches TMPRSS2 center upon its role as a key driver of viral entry for both coronaviruses and influenza viruses. Several treatment techniques have been investigated to reduce TMPRSS2 activity, ranging from direct inhibitors to transcriptional regulatory approaches.

#### 4.2.1. Direct Inhibitors

In vitro, direct inhibition of TMPRSS2 using small-molecule inhibitors such as camostat mesylate, an orally available serine protease inhibitor, effectively blocked SARS-CoV-2 entrance. However, its fast metabolism in the circulation restricts its bioavailability in the airway, indicating that topical application may be more beneficial [128]. Another interesting possibility is MM3122, a new TMPRSS2 inhibitor with better activity than current inhibitors like camostat and nafamostat [48]. When administered to the airway, camostat mesylate successfully inhibited SARS-CoV-2 entry, implying a potential for pre- or postexposure prophylaxis. MM3122 and DRP-104, a prodrug of 6-diazo-5-oxo-l-norleucine (DON), have shown potent antiviral effects and improved stability for preclinical studies with intriguing pharmacokinetics and safety profiles and displayed significant antiviral activity against SARS-CoV-2 and MERS-CoV in human lung epithelial cells [48]. MM3122 exhibited subnanomolar potency against TMPRSS2, matriptase, and hepsin. It has shown vigorous antiviral actions against SARS-CoV-2, in both inhibiting viral replication and preserving cell viability in human lung epithelial cells; these compounds reported efficacy against the EG.5.1 variant. In vivo, the compound offers significant protection against COVID-19 in a mouse model, reducing weight loss, lung congestion, and associated pathology with prophylactic and therapeutic dosing. DRP-104 demonstrated better TMPRSS2 inhibition via synergistic binding of S1/S1′ subdomains and conferred structural stability with a superior thermodynamic profile compared to alternate inhibitors. Both these agents exhibit strong antiviral activities in cell viability assays and also in mouse models exhibiting significant protective effects against SARS-CoV-2 infection, as evidenced by reduced bodyweight loss and lung involvement in mice [129, 130]. While primarily targeting the virus, some monoclonal antibodies can also interfere with TMPRSS2-mediated activities [131]. Benzylsulfonyl-D-arginine-proline-4-amidinobenzylamide (BAPA) acts by inhibiting the TMPRSS2 and HAT to prevent cleavage of HA. It has been demonstrated that this compound effectively inhibits virus spreading in TMPRSS2-expressing human airway epithelial cells, which led to at least 2 logs reduction of virus titers [132]. BAPA with oseltamivir combination therapies is a better way to inhibit influenza virus replication. BAPA works by blocking TMPRSS2; oseltamivir is a neuraminidase inhibitor. The synergistic effect of the drugs significantly reduces the amount needed to achieve strong antiviral effects compared to using either drug alone [132]. Substrate analog inhibitors potently inhibit TMPRSS2 with a 4-amidinobenzylamide moiety at the P1 position. Some analogs have IC50 values in the low nanomolar range, and sulfamoyl derivatives show even greater potency [120].

#### 4.2.2. Transcriptional Regulation

Another feasible option is to target the transcriptional control of TMPRSS2 with androgen receptor (AR) inhibitors. They have already been licensed for prostate cancer treatment and demonstrated to suppress the expression of TMPRSS2 and ACE2, hence limiting SARS-CoV-2 infection. Bromodomain and extra-terminal domain (BET) inhibitors currently being developed for other applications could also be repurposed for COVID-19 treatment. Epidemiological findings show that inhibiting androgen signaling protects against COVID-19 [120].

#### 4.2.3. Combinational Therapies

Furthermore, combining TMPRSS2 with furin inhibitors increases antiviral efficacy against SARS-CoV-2, indicating a synergistic approach for more effective treatment [77, 115].

#### 4.2.4. Natural Products

Ginsenosides, a natural chemical, show potential; however, extensive research in particular patient groups, particularly patients with lung adenocarcinoma (LUAD) as well as those receiving anti-PD-1 therapy, where TMPRSS2 expression is linked with a better prognosis [133]. Research on various phytopharmaceuticals also found that some natural compounds like emodin, luteolin, nicotianamine, and andrographolide could possess potent antiviral activity by targeting TMPRSS2 and furin, two enzymes involved in the viral entry process [78]. Inhibition of TMPRSS2 may affect immune responses. At the same time, it may lessen disease severity by reducing viral entrance while posing dangers of immunological dysregulation [133]. Reduced expression of TMPRSS2 has been linked to poorer outcomes in lung cancer and COVID-19 patients, indicating its dual relevance as a target for therapy and prognosis marker. Continued research into TMPRSS2 inhibition is critical for creating successful antiviral therapies and better patient management measures.

#### 4.2.5. Allosteric Inhibitors

Bromhexine acts as an allosteric inhibitor, binding competitively for TMPRSS2 at the same site as N-terminal Ile-256, and peptidomimetic inhibitors were identified via molecular docking and custom-synthesized using an electrophilic serine trap to target the catalytic site of TMPRSS2, demonstrating effectiveness against multiple SARS-CoV-2 variants in vitro with clinically significant off-target selectivity and stability in blood serum and plasma. They also potently neutralize SARS-CoV-2 spike-driven false virus entry and genuine infections, covering all known variants, including delta and omicron variants [134, 135].

Known inhibitors, such as camostat and nafamostat, are in clinical development and have shown promise in lowering viral loads and SARS-CoV-2 infections. Camostat mesylate resulted in less time for clinical improvement. Compared to lopinavir/ritonavir, it reduced the requirement for mechanical ventilation or death while having no significant impact on 29-day mortality rates [136]. Camostat, another clinically authorized medication, suppresses TMPRSS2 and is rapidly metabolized into (4-(4-guanidinobenzoyloxy) phenylacetic acid (GBPA), which maintains inhibitory actions on TMPRSS2 [137]. However, their impact on clinical results is inconsistent. Preclinical investigations have shown that new inhibitors like MM3122 and repurposed medications like α-ketoamide inhibitor (RAY1216) effectively reduce SARS-CoV-2 infection in vitro and in vivo [48, 138]. Nafamostat is a highly effective inhibitor that has demonstrated remarkable efficacy against SARS-CoV-2 by inhibiting S protein's cleavage by TMPRSS2. This mechanism generates a permanent covalent bond with TMPRSS2, inhibiting its enzymatic activity [139].

A detailed comparison between the two drugs is shown in Table 2.

These inhibitors prevent viral entrance and limit viral replication and pathogenicity. Research suggests that treatment with these inhibitors can considerably reduce viral loads in infected cells and improve results in COVID-19 animal models [95]. Combination therapy inhibiting TMPRSS2 along with additional proteases has demonstrated synergistic effects in lowering viral loads. Dressing TMPRSS2 and cathepsin B dramatically reduced SARS-CoV-2 viral load in human iPS cells, indicating a synergistic impact [139]. Downregulating TMPRSS2 expression via diverse methods provides alternate approaches to fighting viral infections. The therapeutic promise of TMPRSS2 inhibitors extends beyond COVID-19, with the potential for broad-spectrum efficacy against multiple cancers, as shown in Table 3. Future studies should optimize dose regimens, evaluate safety profiles, and investigate combination medicines to improve therapeutic efficacy against viral pathogenesis.

### 4.3. TMPRSS2 Inhibition Using Targeted Antiviral Therapy

In vitro research has shown that peptidomimetic drugs can efficiently limit the action of TMPRSS2 in cell cultures, as evident through the chemical N-0385, which has a significant reduction in SARS-CoV-2 infection by impeding the TMPRSS2-mediated cleavage of S protein [79]. Experiments in animal models supported the findings, providing solid in vivo evidence for the efficacy of TMPRSS2 inhibitors [79]. In humanized mouse models expressing ACE2, administering N-0385 before or during viral exposure significantly decreased mortality and morbidity associated with SARS-CoV-2 infection. The therapeutic window poses a substantial difficulty regarding the delivery time, as N-0385 demonstrated maximum efficacy when administered quickly after exposure or during the early stages of infection [148]. Key findings reveal that modified serpin inhibitors, particularly those targeting TMPRSS2, have effectively prevented ARS-CoV-2 entry and replication in vitro [149]. Antisense oligomers, particularly peptide-conjugated morpholino oligomers, targeting TMPRSS2 mRNA have shown potential in reducing influenza virus titers by blocking the proteolytic activation of influenza virus HA, dramatically reducing viral titers in human airway cells.

Medication repurposing efforts have revealed FDA-approveddrugs like lopinavir and valrubicin as prospective dual inhibitors of TMPRSS2 and ACE2 [150]. High-throughput screening has found new small chemical inhibitors, while established inhibitors such as camostat and nafamostat are currently in clinical studies, proving efficacy in preventing viral entry [151]. Future research into TMPRSS2 inhibition confronts both problems and opportunities, which might considerably impact therapeutic techniques. Improved selectivity is critical, finding inhibitors that target only TMPRSS2, while sparing other serine proteases may improve treatment efficacy and reduce side effects. Furthermore, combination therapies are worth investigating since combining TMPRSS2 inhibitors with other antiviral medications may produce synergistic effects, increasing treatment outcomes for COVID-19 and other viral infections. Furthermore, mechanistic studies are critical; continued research into the molecular mechanisms that regulate TMPRSS2 activity will aid in developing and improving more effective medications. To summarize, targeting TMPRSS2 is a promising method for antiviral therapy against SARS-CoV-2 and possibly other respiratory viruses. Continued research efforts are required to translate these discoveries into helpful clinical therapies.

### 4.4. Drug Repurposing and Development of Novel TMPRSS2 Inhibitors

Drug repurposing, or repositioning, identifies novel therapeutic applications for existing medications, greatly expediting development by leveraging preexisting safety and efficacy data. This technique minimizes development time and costs because current medications have undergone rigorous testing. Furthermore, knowing pharmacokinetic patterns enables more efficient clinical trial design. The urgent need for effective treatments emphasized by the COVID-19 pandemic has prompted research into TMPRSS2 inhibitors as potential antiviral medicines against SARS-CoV-2 [61]. This has resulted in repurposing already existing medications that could suppress TMPRSS2 activity. Virtual screening and structural modeling were utilized to find possible TMPRSS2 inhibitors. Existing medicines, like camostat and nafamostat, are being tested in clinical studies for their efficacy as TMPRSS2 inhibitors. Camostat mesylate, initially developed for pancreatitis, and nafamostat have exhibited efficiency in blocking TMPRSS2-mediatedSARS-CoV-2 entry, with continuing clinical trials examining its effectiveness against COVID-19, demonstrating potential antiviral characteristics against the virus [152].

Computational studies have identified additional candidates for repurposing, including benzquercin, alvimopan, and various anti-HCoV drugs [150, 153–155]. High-throughput screening tests have resulted in the discovery of new peptidomimetic inhibitors, backed by structural analysis and molecular docking approaches that target specific TMPRSS2 residues for selective inhibition. Virtual screening and biochemical assays have discovered new noncovalent TMPRSS2 inhibitors, such as debrisoquine, that block SARS-CoV-2 infectivity in cellular models [151]. Homology modeling and virtual screening have discovered natural products such vicenin-2, neohesperidin, naringin, and rhoifolin as possible TMPRSS2 inhibitors [156]. Future studies should look into combination therapy, viral variants, and clinical studies to determine the safety and efficacy of discovered inhibitors.

Repurposing of current licensed medications addressing homologous serine proteases is another possible path. Several investigational or experimental treatments may inhibit TMPRSS2, such as those targeting S1A serine proteases, which share a high identity with TMPRSS2 in crucial subsites [157]. Lumacaftor and ergotamine were discovered based on virtual screening followed by molecular dynamics simulation studies. They were also determined to be good binders with good binding stability with TMPRSS2, so they are considered potent in the future [158]. Through e-pharmacophore-based screening, molecular docking, and molecular dynamic simulations, three compounds, namely Z126202570, Z46489368, and Z422255982, were identified. They appeared promising and displayed stable binding interactions toward TMPRSS2, which required more in vitro and clinical analysis [48]. Virtual screening has also identified repurposed drugs like rubitecan and loprazolam as potential TMPRSS2 inhibitors. Among these, antithrombin (AT), an endogenous serine protease inhibitor, has appreciable antiviral activity against a broader range of coronaviruses due to inhibition of TMPRSS2 [159]. AT, an endogenous serine protease inhibitor that inhibits TMPRSS2, demonstrated broad-spectrum activity against other coronaviruses [159].

A deep learning-based drug–target interaction model predicts that certain anti-hepatitis C virus (HCV) medicines, like ombitasvir, daclatasvir, and paritaprevir, have a substantial affinity for TMPRSS2 and could potentially be repurposed for COVID-19 treatment [77]. Combining TMPRSS2 inhibitors with additional antiviral medicines or protease inhibitors has revealed more effective antiviral efficacy against SARS-CoV-2 than any single inhibitor alone [77].

### 4.5. Enhancing Public Health Outcomes by Targeting TMPRSS2

Inhibiting TMPRSS2 is a promising treatment method for preventing the entry and spread of influenza viruses and coronaviruses, particularly SARS-CoV-2. Thus, reducing TMPRSS2 activity may limit disease transmission rates and severity among populations [160]. This strategy may be particularly effective in high-risk groups, such as elderly persons or individuals with comorbidities. Understanding the link between TMPRSS2 expression and testosterone signaling may provide insights into gender discrepancies reported in COVID-19 susceptibility and outcomes, particularly among male patients [64]. Studies have indicated that while camostat mesylate, a TMPRSS2 inhibitor, did not significantly improve clinical outcomes for hospitalized COVID-19 patients, it was related to decreased viral loads [161]. Alternative methods, such as peptide-conjugated phosphorodiamidate morpholino oligomers (PPMO), efficiently inhibit the activation of viral proteins in human airway cells [162].

Natural substances, such as ginsenosides, show promise as TMPRSS2 inhibitors but require careful investigation due to potential interactions with other medications [68]. Animal studies show that TMPRSS2 deletion mice have less severe lung damage during viral infections, highlighting its potential as a therapeutic target. TMPRSS2 is involved in respiratory viruses and prostate cancer, providing prospects for treatment techniques targeting viral infections and malignancies [46]. Modulating TMPRSS2 expression or activity may improve therapeutic outcomes for patients confronting these combination problems [96]. Incorporating these discoveries into public health initiatives, such as vaccination campaigns and targeted antiviral medicines, allows healthcare systems to manage outbreaks better and improve population health outcomes from viral respiratory tract infections.

## 5. Future Directions and Research Opportunities

Despite knowledge of TMPRSS2 role in SARS-CoV-2 entry into host cells, substantial gaps in understanding prevent the development of effective therapeutic strategies, especially concerning the specific molecular mechanisms that govern TMPRSS2 enzymatic activity and interactions with both viral and host proteins. Key areas of ambiguity include the molecular mechanisms underpinning the enzymatic activity of TMPRSS2 and its interactions with viral and host proteins, particularly its nonprotease domains and their regulatory implications. Research on TMPRSS2 verifies its proteolytic function. However, further investigation is needed to determine its substrate selectivity and enzymatic activity. Although studies indicate that TMPRSS2 interacts with viral proteins and host factors, direct evidence of these interactions and their impact on viral infectivity is limited [76]. Recent findings suggest that nonprotease domains of TMPRSS2 may influence its function, with phosphorylation of specific residues in its cytosolic tail potentially modulating activity; however, the detailed mechanisms are still underexplored use homology modeling and high-throughput screening to find possible candidates, which could eventually lead to the development of novel drugs that can efficiently suppress TMPRSS2 function [110]. Fragment-based drug design (FBDD) studies have indicated potential in developing effective TMPRSS2 inhibitors. However, the move from in vitro to clinical applications presents substantial obstacles. Existing drugs, such as camostat mesylate, have been repurposed as TMPRSS2 inhibitors; nevertheless, extensive assessments of their efficacy against various respiratory viruses are required to validate wider therapeutic use [95]. The structural and molecular interactions between TMPRSS2 and the SARS-CoV-2 spike protein are still unknown. Identifying TMPRSS2 cleavage sites on the spike protein and modeling their interactions can give insights into viral entrance mechanisms [163]. Genetic studies have revealed quantitative trait loci (eQTLs) associated with TMPRSS2 expression, revealing differences in sensitivity to viral infections among groups. While the consequences of eQTLs on disease susceptibility are substantial, greater study is required to understand how genetic variation affects TMPRSS2 expression and COVID-19 results in distinct populations [164]. TMPRSS2 expression, affected by sex hormones, could contribute to the more serious nature of COVID-19 in men; nevertheless, comparable expression indices in lung regions across genders imply that other variables most likely affect disease severity [165].

TMPRSS2 and ACE2 co-express in bronchial transient secretory and nasal goblet secretory cells, which renders them more vulnerable to SARS-CoV-2 infection. Understanding how varying levels of expression correspond with the extent of the disease may aid in stratifying patients for specific therapy methods [164]. Inhibitors such as camostat and nafamostat are now undergoing clinical investigations. However, the effectiveness of these drugs appears uncertain, demanding additional research regarding their wider potential against other respiratory viruses. Furthermore, genetic investigations demonstrate that TMPRSS2 expression levels could vary across populations in general, influencing susceptibility to COVID-19; however, these outcomes require further investigation. Prospective studies ought to employ an interdisciplinary approach to better comprehend this protease's structure–function relationship, combining knowledge from molecular biology, computational modeling, and clinical information to establish an extensive framework for developing therapies.

TMPRSS2, a transmembrane serine protease, has become a promising therapeutic target due to its significant role in various disorders, notably COVID-19. Prospective studies must emphasize the development of novel TMPRSS2 inhibitors, as the present pharmacological alternatives, including camostat and nafamostat, are hampered by the absence of a crystal structure for TMPRSS2, greatly hampering rational medicinal product development attempts [166]. Recent advances in high-throughput and virtual screening approaches have found several intriguing inhibitors, which require additional confirmation through both in vitro and in vivo research [124, 149, 155]. Moreover, structural modeling can increase our understanding of the TMPRSS2 binding sites [149, 156]. At the same time, comprehensive examinations of its expression throughout multiple tissues, particularly in the respiratory system, may yield a beneficial understanding of its regulatory processes and consequences for the pathogenesis of viruses. Understanding TMPRSS2 interactions with host factors like ACE2 is critical for developing effective combination therapy. Furthermore, repurposing pharmacological medicines that block TMPRSS2 can quickly solve emergent viral threats [167]. In conclusion, a multidisciplinary approach that includes molecular biology, pharmacology, immunology, and clinical research is required to fully understand TMPRSS2's varied role and aid the development of effective therapeutics for COVID-19 and other respiratory viral infections.

### 5.1. Implications for Vaccine Development and Antiviral Strategies

Significant findings suggest that TMPRSS2 serves an integral part in the cleavage and activation of vital viral proteins, like the HA protein in influenza viruses and the spike (S) protein in coronaviruses, that are essential for viral entry into host cells and subsequent transmission [160]. Inhibiting TMPRSS2 has been identified as a successful antiviral strategy, as specific inhibitors may effectively block the replication of these viral proteins, thus substantially lowering infectiousness and propagation, which has been supported by investigations that involve both influenza viruses and coronaviruses, including SARS-CoV-2 and MERS-CoV [61, 123]. Certain inhibitors, including benzoselenoxanthenes and PPMO, have demonstrated efficacy in downregulating TMPRSS2 expression and preventing viral propagation in vitro [123, 162]. Also, animal models that lack TMPRSS2 reveal a substantially decreased degree of lung pathology and spread of the virus after infections with SARS-CoV and MERS-CoV, emphasizing the essential function of this protease in viral pathogenesis and the potential benefits of therapies targeting TMPRSS2 [46]. The broad-spectrum antiviral activity of TMPRSS2 inhibitors goes beyond SARS-CoV-2, indicating that they can be used against diverse coronaviruses and influenza viruses because they inhibit various viral strains [48, 61, 168]. TMPRSS2 is also co-expressed with viral receptors like ACE2 in the human respiratory and gastrointestinal systems. This suggests that targeting TMPRSS2 may reduce the viral entrance and spread inside these tissues [55, 86]. To summarize, strategically aimed at TMPRSS2 represents a multifaceted approach to the development of antiviral therapies and vaccines for both influenza viruses and coronaviruses; inhibitors that inhibit this protease not only prevent the activation of critical viral proteins but also have a broad-spectrum potential as helpful candidates for addressing both current and future viral threats.

## 6. Conclusion

TMPRSS2 is a transmembrane serine protease that activates viral S proteins required for viruses such as SARS-CoV-2 to enter host cells. Its levels of expression change with age and health status, affecting an individual's vulnerability to infection. This heterogeneity highlights the significance of TMPRSS2 in understanding how diverse populations may respond to viral threats. TMPRSS2 is a transmembrane serine protease that activates viral S proteins required for viruses such as SARS-CoV-2 to enter host cells. Its levels of expression change with age and health status, affecting an individual's vulnerability to infection. This heterogeneity highlights the significance of TMPRSS2 in understanding how diverse populations may respond to viral threats. In cells that express TMPRSS2, SARS-CoV-2 can enter quickly, whereas, in the absence of it, the virus prefers slower endosomal pathways. This demonstrates the effectiveness of targeting TMPRSS2 for antiviral therapies. Recent advances in TMPRSS2 inhibitors, such as camostat mesylate and new drugs like MM3122, have shown promise in lowering viral loads in preclinical investigations.

Furthermore, targeting TMPRSS2 may apply to other respiratory diseases besides SARS-CoV-2, such as other influenza viruses. The ongoing clinical trials evaluating the safety and efficacy of TMPRSS2 inhibitors are critical to transferring these discoveries into clinical practice. Developing tailored antivirals that decrease TMPRSS2 function could be a huge step forward in fighting respiratory viral infections. This strategy seeks to reduce the effects of current viral threats. It prepares for future pandemics by laying the groundwork for quick treatment responses.

## Figures and Tables

**Table 1:** List of current therapeutic strategies for respiratory viral infections.

Viral target	Treatment type	Therapeutic strategy	Mechanism of action	Efficacy and limitations	Side effects	Reference
Influenza	Antiviral	Baloxavir marboxil	Inhibits endonuclease activity, blocking viral mRNA synthesis	Single-dose treatment reduces symptoms; limited efficacy in severe cases; resistance observed	Diarrhea, headache, nausea	[25]
		Oseltamivir	Inhibits neuraminidase, preventing viral release	Effective within 48 h of symptoms; limited efficacy in severe cases	Nausea, vomiting, headache, rare neuropsychiatric events	[26]
		Peramivir	Inhibits neuraminidase, preventing viral release	Reduces symptom duration if administered early; limited efficacy in severe cases	Nausea, diarrhea, potential renal toxicity	[27]
		Zanamivir	Inhibits neuraminidase; inhalation route	Effective if given early; limited by inhalation route, especially for respiratory patients	Cough, bronchospasm in asthma/COPD patients	[28]
	Prophylactic	Influenza vaccine	Stimulates immune response against influenza strains	Reduces risk; efficacy depends on strain match; not effective for COVID-19	Localized soreness, fever, low-grade malaise	[29]

SARS-CoV-2 (COVID-19)	Antiviral	Favipiravir	Inhibits viral RNA polymerase	Moderate efficacy in mild cases; less effective in severe cases	Hyperuricemia, potential teratogenicity	[30]
		Molnupiravir	Causes lethal mutagenesis in SARS-CoV-2 RNA	Reduces hospitalization rates; concerns about mutagenic potential and long-term safety	Diarrhea, headache, potential teratogenic effects	[31]
		Remdesivir	Inhibits viral RNA-dependent RNA polymerase	Reduces recovery time in hospitalized patients; limited benefit in mild cases; unsuitable for oral use	Hepatotoxicity, renal toxicity, infusion reactions, nausea	[32]
		Ritonavir	Inhibits SARS-CoV-2 main protease, preventing replication	Reduces hospitalization risk in high-risk patients when given early; potential resistance concerns	Diarrhea, hypertension, liver enzyme elevation, drug interactions	[33]
	Monoclonal antibody	Sotrovimab	Binds to spike protein, blocking viral entry	Reduces hospitalization/death if given early; limited by availability and resistance	Infusion reactions, allergic reactions	[34]
	Immunomodulatory	Convalescent plasma	Provides antibodies from recovered patients	Mixed efficacy; better outcomes in early disease stages and specific populations	Risk of transfusion reactions, limited antibody efficacy	[35]
		Dexamethasone	Glucocorticoid reducing inflammation via cytokine inhibition	Reduces mortality in severe cases; not recommended for mild cases	Hyperglycemia, increased infection risk	[36]
		Tocilizumab	IL-6 receptor antagonist, reducing inflammation	Improves outcomes in severe COVID-19 with systemic inflammation; efficacy depends on timing and patient factors	Risk of infections, gastrointestinal perforation	[37]
	Prophylactic	COVID-19 vaccines (mRNA, viral vector)	Induces immune response via viral spike protein antigens	Highly effective in reducing infections and severe outcomes, especially short term	Injection site pain, fever, rare myocarditis (mRNA vaccines)	[38, 39]

**Table 2:** Comparative analysis of TMPRSS2 inhibitors (camostat and nafamostat).

Properties	Camostat	Nafamostat
Class	Serine protease inhibitor	Serine protease inhibitor
Structure^∗^	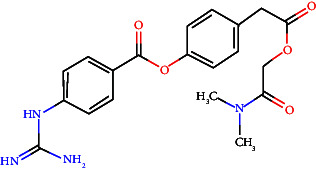	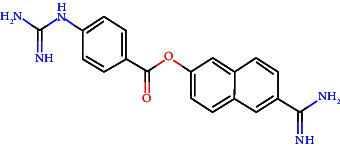
Mechanism of action	Serine protease inhibitor that prevents SARS-CoV-2 entry by inhibiting TMPRSS2 activity.	Serine protease inhibitor with broader activity that also inhibits TMPRSS2 to block viral entry.
Efficacy	IC_50_ = 142 ± 31 nM; effective in reducing viral entry.	IC_50_ = 55 ± 7 nM; more potent than camostat in inhibiting TMPRSS2.
Clinical trial phase	Approved for chronic pancreatitis; used in COVID-19 studies.	Phase 3 clinical trials for COVID-19.
Route of administration	Oral administration	Intravenous administration
Duration of action	Short-acting; requires multiple doses per day	Short-acting; used in acute care settings due to rapid action
Safety profile	Increased risk of serious adverse events: Abdominal pain, elevated liver enzymes.	Hyperkalemia, gastrointestinal issues (nausea, diarrhea), potential bleeding risks.
Additional uses	• Overall, it is ineffective in improving clinical outcomes for COVID-19; there is a higher risk of serious adverse events compared to a placebo. • Primarily used for treating pancreatitis and chronic inflammation.	• Exhibits anticoagulant and anti-inflammatory properties; may improve mucus clearance and has a rapid action profile. • Primarily used as an anticoagulant in acute pancreatitis and other inflammatory conditions.
Limitations	Limited efficacy data in severe COVID-19 cases; oral bioavailability limitations	Limited data in outpatient settings; efficacy largely tested in controlled hospital environments
Potential for resistance	Low potential for resistance development	Low potential for resistance; however, rapid clearance may limit the therapeutic window

^∗^The chemical structure images were taken from ChEMBL (https://www.ebi.ac.uk/chembl/).

**Table 3:** Clinical trials of various TMPRSS2 inhibitors: ongoing and completed studies.

Primary drugs	Mechanism of action	Indication(s)	Trial ID^∗^	Phase of trial	Trial status	References
Abiraterone, proxalutamide	Abiraterone blocks CYP17A1, lowering androgen levels and reducing androgen receptor (AR) signaling. This inhibition of androgen synthesis indirectly impacts the expression of the TMPRSS2–ERG fusion protein expressed in prostate cancer	Prostate cancer	700303948	Phase III	Active, no longer recruiting	[140, 141]

Exemestane, fulvestrant, letrozole, proxalutamide	Exemestane and letrozole are steroidal aromatase inhibitor acting via the AR pathway and prevents the conversion of androgens to estrogens. Fulvestrant, a selective estrogen receptor degrader (SERD), binds to the estrogen receptor (ER) with high affinity, blocking its function and triggering its degradation. By inhibiting estrogen and its signaling these drugs are effective in treating breast cancers.	Advanced breast cancer, HER2-negative breast cancer	700324094	Phase I	Active, no longer recruiting	[142–144]

Proxalutamide	Proxalutamide is an androgen receptor antagonist that modulates AR signaling, influencing TMPRSS2 expression. Since androgen receptor signaling can upregulate TMPRSS2, it may increase cellular susceptibility to SARS-CoV-2 infection. By inhibiting AR signaling, proxalutamide reduces TMPRSS2 expression, potentially limiting SARS-CoV-2 spike protein activation and viral entry into host cells. Additionally, by blocking AR signaling, proxalutamide suppresses TMPRSS2-driven processes, such as cancer cell invasion and metastasis.	Adenocarcinoma, bone metastases, cancer metastases, prostate cancer	700334843	Phase II	Completed	[145–147]
		Prostate cancer	700285876	Phase I	Completed	
			700306106	Phase II	Completed	
			700343656	Phase II	Recruiting	
			700274346	Phase I/II	Completed	
			700303951	Phase III	Recruiting	
		Advanced breast cancer, triple-negative breast cancer	700304009	Phase I	Completed	
		Triple-negative breast cancer	700304007	Phase I	Planning	
		COVID 2019 respiratory infections	700334091	Clinical phase unknown	Completed	
			700333116	Phase III	Completed	
			700334092	Phase III	Recruiting	
			700334750	Phase III	Completed	
			700341621	Phase III	Discontinued	
			700336603	Phase III	Discontinued	
			700356278	Phase II	Recruiting	
			700336323	Phase III	Discontinued	
			700336129	Phase III	Discontinued	
			700328017	Phase II	Completed	
			700345056	Phase III	Completed	

^∗^Data about trials were obtained from AdisInsight (https://adisinsight.springer.com/).

## Data Availability

Data sharing is not applicable as no new data were generated.

## References

[1] Shi T., Arnott A., Semogas I. (2020). The Etiological Role of Common Respiratory Viruses in Acute Respiratory Infections in Older Adults: A Systematic Review and Meta-Analysis. *The Journal of Infectious Diseases*.

[2] Zhou F., Wang Y., Liu Y. (2019). Disease Severity and Clinical Outcomes of Community-Acquired Pneumonia Caused by Non-Influenza Respiratory Viruses in Adults: A Multicentre Prospective Registry Study From the CAP-China Network. *European Respiratory Journal*.

[3] Cates J., Lucero-Obusan C., Dahl R. M. (2020). Risk for In-Hospital Complications Associated With COVID-19 and Influenza—Veterans Health Administration, United States, October 1, 2018–May 31, 2020. *MMWR Morb Mortal Wkly Rep*.

[4] Arabi Y. M., Fowler R., Hayden F. G. (2020). Critical Care Management of Adults With Community-Acquired Severe Respiratory Viral Infection. *Intensive Care Medicine*.

[5] Mochan E., Sego T. J. (2023). Mathematical Modeling of the Lethal Synergism of Coinfecting Pathogens in Respiratory Viral Infections: A Review. *Microorganisms*.

[6] Robinson C. P., Busl K. M. (2020). Neurologic Manifestations of Severe Respiratory Viral Contagions. *Critical Care Explorations*.

[7] Gallo V., Arienzo A., Iacobelli S., Iacobelli V., Antonini G. (2022). Gal-3BP in Viral Infections: An Emerging Role in Severe Acute Respiratory Syndrome Coronavirus 2. *International Journal of Molecular Sciences*.

[8] Di Maio V. C., Scutari R., Forqué L. (2024). Presence and Significance of Multiple Respiratory Viral Infections in Children Admitted to a Tertiary Pediatric Hospital in Italy. *Viruses*.

[9] Ahmed R., Anam K., Ahmed H. (2023). Development of Galectin-3 Targeting Drugs for Therapeutic Applications in Various Diseases. *International Journal of Molecular Sciences*.

[10] Caniglia J. L., Asuthkar S., Tsung A. J., Guda M. R., Velpula K. K. (2020). Immunopathology of Galectin-3: an Increasingly Promising Target in COVID-19. *F1000Res*.

[11] Choi E. J., Wu W., Cong X. (2021). Broad Impact of Exchange Protein Directly Activated by Camp 2 (Epac2) on Respiratory Viral Infections. *Viruses*.

[12] Choi E. J., Ren Y., Chen Y. (2018). Exchange Proteins Directly Activated by cAMP and Their Roles in Respiratory Syncytial Virus Infection. *Journal of Virology*.

[13] Aviani J. K., Halim D., Soeroto A. Y., Achmad T. H., Djuwantono T. (2021). Current Views on the Potentials of Convalescent Plasma Therapy (CPT) as Coronavirus Disease 2019 (COVID-19) Treatment: A Systematic Review and Meta-Analysis Based on Recent Studies and Previous Respiratory Pandemics. *Reviews in Medical Virology*.

[14] Yousefi B., Valizadeh S., Ghaffari H., Vahedi A., Karbalaei M., Eslami M. (2020). A Global Treatments for Coronaviruses Including COVID-19. *Journal of Cellular Physiology*.

[15] Jean S.-S., Lee P.-I., Hsueh P.-R. (2020). Treatment Options for COVID-19: The Reality and Challenges. *Journal of Microbiology, Immunology, and Infection*.

[16] Chen C.-P., Lin Y.-C., Chen T.-C. (2020). A Multicenter, Randomized, Open-Label, Controlled Trial to Evaluate the Efficacy and Tolerability of Hydroxychloroquine and a Retrospective Study in Adult Patients With Mild to Moderate Coronavirus Disease 2019 (COVID-19). *PLoS One*.

[17] Réa-Neto Á., Bernardelli R. S., Câmara B. M. D., Reese F. B., Queiroga M. V. O., Oliveira M. C. (2021). An Open-Label Randomized Controlled Trial Evaluating the Efficacy of Chloroquine/Hydroxychloroquine in Severe COVID-19 Patients. *Scientific Reports*.

[18] Self W. H., Semler M. W., Leither L. M. (2020). Effect of Hydroxychloroquine on Clinical Status at 14 Days in Hospitalized Patients With COVID-19. *JAMA*.

[19] Kashour Z., Kashour T., Gerberi D., Tleyjeh I. M. (2021). Mortality, Viral Clearance, and Other Clinical Outcomes of Hydroxychloroquine in COVID‐19 Patients: A Systematic Review and Meta‐Analysis of Randomized Controlled Trials. *Clinical and Translational Science*.

[20] Ulrich R. J., Troxel A. B., Carmody E. (2020). Treating COVID-19 with Hydroxychloroquine (TEACH): A Multicenter, Double-Blind Randomized Controlled Trial in Hospitalized Patients. *Open Forum Infectious Diseases*.

[21] Amani B., Khanijahani A., Amani B. (2021). Hydroxychloroquine Plus Standard of Care Compared with Standard of Care Alone in COVID-19: A Meta-Analysis of Randomized Controlled Trials. *Scientific Reports*.

[22] Hernandez A. V., Roman Y. M., Pasupuleti V., Barboza J. J., White C. M. (2020). Hydroxychloroquine or Chloroquine for Treatment or Prophylaxis of COVID-19: A Living Systematic Review. *Annals of Internal Medicine*.

[23] Ryabkova V. A., Churilov L. P., Shoenfeld Y. (2021). Influenza Infection, SARS, MERS and COVID-19: Cytokine Storm—the Common Denominator and the Lessons to Be Learned. *Clinical Immunology*.

[24] Khoury M., Cuenca J., Cruz F. F., Figueroa F. E., Rocco P. R. M., Weiss D. J. (2020). Current Status of Cell-Based Therapies for Respiratory Virus Infections: Applicability to COVID-19. *European Respiratory Journal*.

[25] Hayden F. G., Sugaya N., Hirotsu N. (2018). Baloxavir Marboxil for Uncomplicated Influenza in Adults and Adolescents. *New England Journal of Medicine*.

[26] Jefferson T., Jones M., Doshi P., Spencer E. A., Onakpoya I., Heneghan C. J. (2014). Oseltamivir for Influenza in Adults and Children: Systematic Review of Clinical Study Reports and Summary of Regulatory Comments. *BMJ*.

[27] Scott L. J. (2018). Peramivir: A Review in Uncomplicated Influenza. *Drugs*.

[28] Heneghan C. J., Onakpoya I., Thompson M., Spencer E. A., Jones M., Jefferson T. (2014). Zanamivir for Influenza in Adults and Children: Systematic Review of Clinical Study Reports and Summary of Regulatory Comments. *BMJ*.

[29] Grohskopf L. A., Blanton L. H., Ferdinands J. M. (2022). Prevention and Control of Seasonal Influenza with Vaccines: Recommendations of the Advisory Committee on Immunization Practices—United States, 2022–23 Influenza Season. *MMWR. Recommendations and Reports*.

[30] Shiraki K., Daikoku T. (2020). Favipiravir, an Anti-Influenza Drug against Life-Threatening RNA Virus Infections. *Pharmacology & Therapeutics*.

[31] Jayk Bernal A., Gomes da Silva M. M., Musungaie D. B. (2022). Molnupiravir for Oral Treatment of Covid-19 in Nonhospitalized Patients. *New England Journal of Medicine*.

[32] Eastman R. T., Roth J. S., Brimacombe K. R. (2020). Correction to Remdesivir: A Review of Its Discovery and Development Leading to Human Clinical Trials for Treatment of COVID-19. *ACS Central Science*.

[33] Zheng Q., Ma P., Wang M. (2023). Efficacy and Safety of Paxlovid for COVID-19: A Meta-Analysis. *Journal of Infection*.

[34] Focosi D., Casadevall A., Franchini M., Maggi F. (2024). Sotrovimab: A Review of Its Efficacy Against SARS-CoV-2 Variants. *Viruses*.

[35] Simonovich V. A., Burgos Pratx L. D., Scibona P. (2021). A Randomized Trial of Convalescent Plasma in Covid-19 Severe Pneumonia. *New England Journal of Medicine*.

[36] The Recovery Collaborative Group (2021). Dexamethasone in Hospitalized Patients With Covid-19. *New England Journal of Medicine*.

[37] Stone J. H., Frigault M. J., Serling-Boyd N. J. (2020). Efficacy of Tocilizumab in Patients Hospitalized With Covid-19. *New England Journal of Medicine*.

[38] Baden L. R., El Sahly H. M., Essink B. (2021). Efficacy and Safety of the mRNA-1273 SARS-CoV-2 Vaccine. *New England Journal of Medicine*.

[39] Polack F. P., Thomas S. J., Kitchin N. (2020). Safety and Efficacy of the BNT162b2 mRNA Covid-19 Vaccine. *New England Journal of Medicine*.

[40] Falsey A. R., McElhaney J. E., Beran J. (2014). Respiratory Syncytial Virus and Other Respiratory Viral Infections in Older Adults With Moderate to Severe Influenza-Like Illness. *The Journal of Infectious Diseases*.

[41] Scotta M. C., Chakr V. C. B. G., de Moura A. (2016). Respiratory Viral Coinfection and Disease Severity in Children: A Systematic Review and Meta-Analysis. *Journal of Clinical Virology*.

[42] Li Y., Pillai P., Miyake F., Nair H. (2020). The Role of Viral Co-Infections in the Severity of Acute Respiratory Infections Among Children Infected With Respiratory Syncytial Virus (RSV): A Systematic Review and Meta-Analysis. *Journal of Global Health*.

[43] Alhumaid S., Alabdulqader M., Al Dossary N. (2022). Global Coinfections With Bacteria, Fungi, and Respiratory Viruses in Children With SARS-CoV-2: A Systematic Review and Meta-Analysis. *Tropical Medicine and Infectious Disease*.

[44] Vink E., Davis C., MacLean A. (2022). Viral Coinfections in Hospitalized Coronavirus Disease 2019 Patients Recruited to the International Severe Acute Respiratory and Emerging Infections Consortium WHO Clinical Characterisation Protocol UK Study. *Open Forum Infectious Diseases*.

[45] Damasio G. A. C., Pereira L. A., Moreira S. D. R., Duarte dos Santos C. N., Dalla-Costa L. M., Raboni S. M. (2015). Does Virus–Bacteria Coinfection Increase the Clinical Severity of Acute Respiratory Infection?. *Journal of Medical Virology*.

[46] Iwata-Yoshikawa N., Okamura T., Shimizu Y., Hasegawa H., Takeda M., Nagata N. (2019). TMPRSS2 Contributes to Virus Spread and Immunopathology in the Airways of Murine Models after Coronavirus Infection. *Journal of Virology*.

[47] Limburg H., Harbig A., Bestle D. (2019). TMPRSS2 Is the Major Activating Protease of Influenza A Virus in Primary Human Airway Cells and Influenza B Virus in Human Type II Pneumocytes. *Journal of Virology*.

[48] Mahoney M., Damalanka V. C., Tartell M. A. (2021). A Novel Class of TMPRSS2 Inhibitors Potently Block SARS-CoV-2 and MERS-CoV Viral Entry and Protect Human Epithelial Lung Cells. *Proceedings of the National Academy of Sciences of the United States of America*.

[49] Behzadi M. A., Leyva-Grado V. H. (2019). Overview of Current Therapeutics and Novel Candidates against Influenza, Respiratory Syncytial Virus, and Middle East Respiratory Syndrome Coronavirus Infections. *Frontiers in Microbiology*.

[50] Hatesuer B., Bertram S., Mehnert N. (2013). Tmprss2 Is Essential for Influenza H1N1 Virus Pathogenesis in Mice. *PLoS Pathogens*.

[51] Thunders M., Delahunt B. (2020). Gene of the Month: TMPRSS2 (Transmembrane Serine Protease 2). *Journal of Clinical Pathology*.

[52] Paoloni-Giacobino A., Chen H., Peitsch M. C., Rossier C., Antonarakis S. E. (1997). Cloning of the TMPRSS2 Gene, Which Encodes a Novel Serine Protease With Transmembrane, LDLRA, and SRCR Domains and Maps to 21q22.3. *Genomics*.

[53] Chen Y.-W., Lee M.-S., Lucht A. (2010). TMPRSS2, a Serine Protease Expressed in the Prostate on the Apical Surface of Luminal Epithelial Cells and Released into Semen in Prostasomes, Is Misregulated in Prostate Cancer Cells. *American Journal Of Pathology*.

[54] Lucas J. M., Heinlein C., Kim T. (2014). The Androgen-Regulated Protease TMPRSS2 Activates a Proteolytic Cascade Involving Components of the Tumor Microenvironment and Promotes Prostate Cancer Metastasis. *Cancer Discovery*.

[55] Bertram S., Heurich A., Lavender H. (2012). Influenza and SARS-Coronavirus Activating Proteases TMPRSS2 and HAT Are Expressed at Multiple Sites in Human Respiratory and Gastrointestinal Tracts. *PLoS One*.

[56] Tug E., Fidan I., Bozdayi G. (2023). The Relationship Between the Clinical Course of SARS-CoV-2 Infections and ACE2 and TMPRSS2 Expression and Polymorphisms. *Advances in Clinical and Experimental Medicine*.

[57] Muus C., Luecken M. D., Eraslan G. (2021). Single-Cell Meta-Analysis of SARS-CoV-2 Entry Genes Across Tissues and Demographics. *Nature Medicine*.

[58] Zhang H., Kang Z., Gong H. (2020). Digestive System Is a Potential Route of COVID-19: An Analysis of Single-Cell Coexpression Pattern of Key Proteins in Viral Entry Process. *Gut*.

[59] Sun T. K., Huang W. C., Sun Y. W. (2022). Schizophyllum Commune Reduces Expression of the SARS-CoV-2 Receptors ACE2 and TMPRSS2. *International Journal of Molecular Sciences*.

[60] Bertram S., Dijkman R., Habjan M. (2013). TMPRSS2 Activates the Human Coronavirus 229E for Cathepsin-Independent Host Cell Entry and Is Expressed in Viral Target Cells in the Respiratory Epithelium. *Journal of Virology*.

[61] Hoffmann M., Kleine-Weber H., Schroeder S. (2020). SARS-CoV-2 Cell Entry Depends on ACE2 and TMPRSS2 and Is Blocked by a Clinically Proven Protease Inhibitor. *Cell*.

[62] Matsuyama S., Nagata N., Shirato K., Kawase M., Takeda M., Taguchi F. (2010). Efficient Activation of the Severe Acute Respiratory Syndrome Coronavirus Spike Protein by the Transmembrane Protease TMPRSS2. *Journal of Virology*.

[63] Meng B., Abdullahi A., Ferreira I. A. T. M. (2022). Altered TMPRSS2 Usage by SARS-CoV-2 Omicron Impacts Infectivity and Fusogenicity. *Nature*.

[64] Koch J., Uckeley Z. M., Doldan P., Stanifer M., Boulant S., Lozach P. (2021). TMPRSS2 Expression Dictates the Entry Route Used by SARS‐CoV‐2 to Infect Host Cells. *The EMBO Journal*.

[65] Paniri A., Hosseini M. M., Akhavan-Niaki H. (2021). First Comprehensive Computational Analysis of Functional Consequences of TMPRSS2 SNPs in Susceptibility to SARS-CoV-2 Among Different Populations. *Journal of Biomolecular Structure and Dynamics*.

[66] Zarubin A., Stepanov V., Markov A. (2020). Structural Variability, Expression Profile, and Pharmacogenetic Properties of TMPRSS2 Gene as a Potential Target for COVID-19 Therapy. *Genes*.

[67] Cao W., Feng Q., Wang X. (2021). Computational Analysis of TMPRSS2 Expression in Normal and SARS-CoV-2-Infected Human Tissues. *Chemico-Biological Interactions*.

[68] Meng M., Gao R., Liu Z. (2023). Ginsenosides, Potential TMPRSS2 Inhibitors, a Trade-Off Between the Therapeutic Combination for Anti-PD-1 Immunotherapy and the Treatment of COVID-19 Infection of LUAD Patients. *Frontiers in Pharmacology*.

[69] Peters M. C., Sajuthi S., Deford P. (2020). COVID-19–Related Genes in Sputum Cells in Asthma. Relationship to Demographic Features and Corticosteroids. *American Journal of Respiratory and Critical Care Medicine*.

[70] Heurich A., Hofmann-Winkler H., Gierer S., Liepold T., Jahn O., Pöhlmann S. (2014). TMPRSS2 and ADAM17 Cleave ACE2 Differentially and Only Proteolysis by TMPRSS2 Augments Entry Driven by the Severe Acute Respiratory Syndrome Coronavirus Spike Protein. *Journal of Virology*.

[71] Glowacka I., Bertram S., Müller M. A. (2011). Evidence that TMPRSS2 Activates the Severe Acute Respiratory Syndrome Coronavirus Spike Protein for Membrane Fusion and Reduces Viral Control by the Humoral Immune Response. *Journal of Virology*.

[72] Hashimoto R., Sakamoto A., Deguchi S. (2021). Dual Inhibition of TMPRSS2 and Cathepsin B Prevents SARS-CoV-2 Infection in iPS Cells. *Molecular Therapy-Nucleic Acids*.

[73] Sarker J., Das P., Sarker S., Roy A. K., Momen A. Z. M. R. (2021). A Review on Expression, Pathological Roles, and Inhibition of TMPRSS2, the Serine Protease Responsible for SARS-CoV-2 Spike Protein Activation. *Scientifica (Cairo)*.

[74] Böttcher-Friebertshäuser E., Freuer C., Sielaff F. (2010). Cleavage of Influenza Virus Hemagglutinin by Airway Proteases TMPRSS2 and HAT Differs in Subcellular Localization and Susceptibility to Protease Inhibitors. *Journal of Virology*.

[75] Essalmani R., Jain J., Susan-Resiga D. (2022). Distinctive Roles of Furin and TMPRSS2 in SARS-CoV-2 Infectivity. *Journal of Virology*.

[76] Strobelt R., Adler J., Shaul Y. (2023). The Transmembrane Protease Serine 2 (TMPRSS2) Non-Protease Domains Regulating Severe Acute Respiratory Syndrome Coronavirus 2 (SARS-CoV-2) Spike-Mediated Virus Entry. *Viruses*.

[77] Bestle D., Heindl M. R., Limburg H. (2020). TMPRSS2 and Furin are Both Essential for Proteolytic Activation of SARS-CoV-2 in Human Airway Cells. *Life Science Alliance*.

[78] Palit P., Chattopadhyay D., Thomas S., Kundu A., Kim H. S., Rezaei N. (2021). Phytopharmaceuticals Mediated Furin and TMPRSS2 Receptor Blocking: Can It Be a Potential Therapeutic Option for Covid-19?. *Phytomedicine*.

[79] Singh S., O’Reilly S., Gewaid H., Bowie A. G., Gautier V., Worrall D. M. (2022). Reactive Centre Loop Mutagenesis of SerpinB3 to Target TMPRSS2 and Furin: Inhibition of SARS-CoV-2 Cell Entry and Replication. *International Journal of Molecular Sciences*.

[80] Vardhan S., Sahoo S. K. (2022). Virtual Screening by Targeting Proteolytic Sites of Furin and TMPRSS2 to Propose Potential Compounds Obstructing the Entry of SARS-CoV-2 Virus into Human Host Cells. *Journal of Traditional and Complementary Medicine*.

[81] Choudhary S., Silakari O. (2020). Scaffold Morphing of Arbidol (Umifenovir) in Search of Multi-Targeting Therapy Halting the Interaction of SARS-CoV-2 With ACE2 and Other Proteases Involved in COVID-19. *Virus Research*.

[82] Cheng Y.-W., Chao T.-L., Li C.-L. (2020). Furin Inhibitors Block SARS-CoV-2 Spike Protein Cleavage to Suppress Virus Production and Cytopathic Effects. *Cell Reports*.

[83] Liu Y.-M., Shahed-Al-Mahmud Md., Chen X. (2020). A Carbohydrate-Binding Protein From the Edible Lablab Beans Effectively Blocks the Infections of Influenza Viruses and SARS-CoV-2. *Cell Reports*.

[84] Zeng Q., Langereis M. A., van Vliet A. L. W., Huizinga E. G., de Groot R. J. (2008). Structure of Coronavirus Hemagglutinin-Esterase Offers Insight Into Corona and Influenza Virus Evolution. *Proceedings of the National Academy of Sciences*.

[85] Takeda M. (2022). Proteolytic Activation of SARS-CoV-2 Spike Protein. *Microbiology and Immunology*.

[86] Zmora P., Hoffmann M., Kollmus H. (2018). TMPRSS11A Activates the Influenza A Virus Hemagglutinin and the MERS Coronavirus Spike Protein and Is Insensitive against Blockade by HAI-1. *Journal of Biological Chemistry*.

[87] Chan Y. A., Zhan S. H. (2022). The Emergence of the Spike Furin Cleavage Site in SARS-CoV-2. *Molecular Biology and Evolution*.

[88] Romeu A. R. (2023). Probable Human Origin of the SARS-CoV-2 Polybasic Furin Cleavage Motif. *BMC Genom Data*.

[89] Lavie M., Dubuisson J., Belouzard S. (2022). SARS-CoV-2 Spike Furin Cleavage Site and S2′ Basic Residues Modulate the Entry Process in a Host Cell-Dependent Manner. *Journal of Virology*.

[90] Lubinski B., Whittaker G. R. (2023). The SARS-CoV-2 Furin Cleavage Site: Natural Selection or Smoking Gun?. *The Lancet Microbe*.

[91] Xia S., Lan Q., Su S. (2020). The Role of Furin Cleavage Site in SARS-CoV-2 Spike Protein-Mediated Membrane Fusion in the Presence or Absence of Trypsin. *Signal Transduction and Targeted Therapy*.

[92] Liu M., Huang L. Z. X., Smits A. A. (2022). Human-Type Sialic Acid Receptors Contribute to Avian Influenza A Virus Binding and Entry by Hetero-Multivalent Interactions. *Nature Communications*.

[93] Sieben C., Manley S. (2016). Influenza a Viruses Use Multivalent Sialic Acid Clusters for Cell Binding and Receptor Activation. *Biophysical Journal*.

[94] Jackson C. B., Farzan M., Chen B., Choe H. (2022). Mechanisms of SARS-CoV-2 Entry Into Cells. *Nature Reviews Molecular Cell Biology*.

[95] Shapira T., Monreal I. A., Dion S. P. (2022). A TMPRSS2 Inhibitor Acts as a Pan-SARS-CoV-2 Prophylactic and Therapeutic. *Nature*.

[96] Iwata-Yoshikawa N., Kakizaki M., Shiwa-Sudo N. (2022). Essential Role of TMPRSS2 in SARS-CoV-2 Infection in Murine Airways. *Nature Communications*.

[97] Zhang Y., Sun S., Du C. (2022). Transmembrane Serine Protease TMPRSS2 Implicated in SARS-CoV-2 Infection Is Autoactivated Intracellularly and Requires N-Glycosylation for Regulation. *Journal of Biological Chemistry*.

[98] Thaingtamtanha T., Baeurle S. A. (2022). Study of Protease-Mediated Processes Initiating Viral Infection and Cell–Cell Viral Spreading of SARS-CoV-2. *Journal of Molecular Modeling*.

[99] Fraser B. J., Beldar S., Seitova A. (2022). Structure and Activity of Human TMPRSS2 Protease Implicated in SARS-CoV-2 Activation. *Nature Chemical Biology*.

[100] Peacock T. P., Goldhill D. H., Zhou J. (2021). The Furin Cleavage Site in the SARS-CoV-2 Spike Protein Is Required for Transmission in Ferrets. *Nat Microbiol*.

[101] Kazuya S., Kazuhiko K., Miyuki K., Shutoku M. (2016). Clinical Isolates of Human Coronavirus 229E Bypass the Endosome for Cell Entry. *Journal of Virology*.

[102] Lie L. K., Synowiec A., Mazur J., Rabalski L., Pyrć K. (2023). An Engineered A549 Cell Line Expressing CD13 and TMPRSS2 Is Permissive to Clinical Isolate of Human Coronavirus 229E. *Virology*.

[103] Liu Y., Qu H.-Q., Qu J., Tian L., Hakonarson H. (2020). Expression Pattern of the SARS-CoV-2 Entry Genes ACE2 and TMPRSS2 in the Respiratory Tract. *Viruses*.

[104] Schwerdtner M., Schmacke L. C., Nave J. (2024). Unveiling the Role of TMPRSS2 in the Proteolytic Activation of Pandemic and Zoonotic Influenza Viruses and Coronaviruses in Human Airway Cells. *Viruses*.

[105] Heindl M. R., Rupp A.-L., Schwerdtner M. (2024). ACE2 Acts as a Novel Regulator of TMPRSS2-Catalyzed Proteolytic Activation of Influenza A Virus in Airway Cells. *Journal of Virology*.

[106] Ciacci Zanella G., Snyder C. A., Arruda B. L. (2023). Pigs Lacking TMPRSS2 Displayed Fewer Lung Lesions and Reduced Inflammatory Response When Infected With Influenza A Virus. *Frontiers in Genome Editing*.

[107] Galloway S. E., Reed M. L., Russell C. J., Steinhauer D. A. (2013). Influenza HA Subtypes Demonstrate Divergent Phenotypes for Cleavage Activation and pH of Fusion: Implications for Host Range and Adaptation. *PLoS Pathogens*.

[108] Lambertz R. L. O., Gerhauser I., Nehlmeier I. (2019). Tmprss2 Knock-Out Mice are Resistant to H10 Influenza A Virus Pathogenesis. *Journal of General Virology*.

[109] Sakai K., Ami Y., Tahara M. (2014). The Host Protease TMPRSS2 Plays a Major Role in In Vivo Replication of Emerging H7N9 and Seasonal Influenza Viruses. *Journal of Virology*.

[110] Bestle D., Limburg H., Kruhl D. (2021). Hemagglutinins of Avian Influenza Viruses Are Proteolytically Activated by TMPRSS2 in Human and Murine Airway Cells. *Journal of Virology*.

[111] Kehdy F. S. G., Pita-Oliveira M., Scudeler M. M. (2021). Human-SARS-CoV-2 Interactome and Human Genetic Diversity: TMPRSS2-Rs2070788, Associated With Severe Influenza, and Its Population Genetics Caveats in Native Americans. *Genetics and Molecular Biology*.

[112] Cheng Z., Zhou J., To K. K.-W. (2015). Identification of TMPRSS2 as a Susceptibility Gene for Severe 2009 Pandemic A(H1N1) Influenza and A(H7N9) Influenza. *Journal of Infectious Diseases*.

[113] Yaghoobi A., Lord J. S., Rezaiezadeh J. S., Yekaninejad M. S., Amini M., Izadi P. (2023). TMPRSS2 Polymorphism (rs12329760) and the Severity of the COVID-19 in Iranian Population. *PLoS One*.

[114] Schönfelder K., Breuckmann K., Elsner C. (2021). Transmembrane Serine Protease 2 Polymorphisms and Susceptibility to Severe Acute Respiratory Syndrome Coronavirus Type 2 Infection: A German Case-Control Study. *Frontiers in Genetics*.

[115] Qiao Y., Wang X.-M., Mannan R. (2021). Targeting Transcriptional Regulation of SARS-CoV-2 Entry Factors ACE2 and TMPRSS2. *Proceedings of the National Academy of Sciences of the United States of America*.

[116] Sakai K., Ami Y., Nakajima N. (2016). TMPRSS2 Independency for Haemagglutinin Cleavage In Vivo Differentiates Influenza B Virus From Influenza A Virus. *Scientific Reports*.

[117] Harbig A., Mernberger M., Bittel L. (2020). Transcriptome Profiling and Protease Inhibition Experiments Identify Proteases That Activate H3N2 Influenza A and Influenza B Viruses in Murine Airways. *Journal of Biological Chemistry*.

[118] Kühn N., Bergmann S., Kösterke N. (2016). The Proteolytic Activation of (H3N2) Influenza A Virus Hemagglutinin Is Facilitated by Different Type II Transmembrane Serine Proteases. *Journal of Virology*.

[119] Tarnow C., Engels G., Arendt A. (2014). TMPRSS2 Is a Host Factor that Is Essential for Pneumotropism and Pathogenicity of H7N9 Influenza A Virus in Mice. *Journal of Virology*.

[120] Meyer D., Sielaff F., Hammami M., Böttcher-Friebertshäuser E., Garten W., Steinmetzer T. (2013). Identification of the First Synthetic Inhibitors of the Type II Transmembrane Serine Protease TMPRSS2 Suitable for Inhibition of Influenza Virus Activation. *Biochemical Journal*.

[121] Abdollahi S., Izadi P. (2021). TMPRSS2 as an Influential Human Gene for COVID-19. *Journal of Human Genetics and Genomics*.

[122] Metzdorf K., Jacobsen H., Greweling-Pils M. C. (2023). TMPRSS2 Is Essential for SARS-CoV-2 Beta and Omicron Infection. *Viruses*.

[123] Shen L.-W., Qian M.-Q., Yu K. (2020). Inhibition of Influenza A Virus Propagation by Benzoselenoxanthenes Stabilizing TMPRSS2 Gene G-Quadruplex and Hence Down-Regulating TMPRSS2 Expression. *Scientific Reports*.

[124] Lukassen S., Chua R. L., Trefzer T. (2020). SARS-CoV-2 Receptor ACE2 and TMPRSS2 Are Primarily Expressed in Bronchial Transient Secretory Cells. *The EMBO Journal*.

[125] Matsuyama S., Nao N., Shirato K. (2020). Enhanced Isolation of SARS-CoV-2 by TMPRSS2-Expressing Cells. *Proceedings of the National Academy of Sciences*.

[126] Lü M., Qiu L., Jia G., Guo R., Leng Q. (2020). Single-cell Expression Profiles of ACE2 and TMPRSS2 Reveals Potential Vertical Transmission and Fetus Infection of SARS-CoV-2. *Aging*.

[127] Gierer S., Bertram S., Kaup F. (2013). The Spike Protein of the Emerging Betacoronavirus EMC Uses a Novel Coronavirus Receptor for Entry, Can Be Activated by TMPRSS2, and Is Targeted by Neutralizing Antibodies. *Journal of Virology*.

[128] Guo W., Porter L. M., Crozier T. W. M. (2022). Topical TMPRSS2 Inhibition Prevents SARS-CoV-2 Infection in Differentiated Human Airway Cultures. *Life Science Alliance*.

[129] Boon A. C. M., Bricker T. L., Fritch E. J. (2024). Efficacy of Host Cell Serine Protease Inhibitor MM3122 Against SARS-CoV-2 for Treatment and Prevention of COVID-19. *Journal of Virology*.

[130] Shakya A., Chikhale R. V., Bhat H. R. (2022). Pharmacoinformatics-Based Identification of Transmembrane Protease Serine-2 Inhibitors From Morus Alba as SARS-CoV-2 Cell Entry Inhibitors. *Molecular Diversity*.

[131] Reuter N., Chen X., Kropff B. (2023). SARS-CoV-2 Spike Protein Is Capable of Inducing Cell–Cell Fusions Independent From Its Receptor ACE2 and This Activity Can Be Impaired by Furin Inhibitors or a Subset of Monoclonal Antibodies. *Viruses*.

[132] Böttcher-Friebertshäuser E., Lu Y., Meyer D. (2012). Hemagglutinin Activating Host Cell Proteases Provide Promising Drug Targets for the Treatment of Influenza A and B Virus Infections. *Vaccine*.

[133] Nazerian Y., Vakili K., Ebrahimi A., Niknejad H. (2021). Developing Cytokine Storm-Sensitive Therapeutic Strategy in COVID-19 Using 8P9R Chimeric Peptide and Soluble ACE2. *Frontiers in Cell and Developmental Biology*.

[134] Sgrignani J., Cavalli A. (2021). Computational Identification of a Putative Allosteric Binding Pocket in TMPRSS2. *Frontiers in Molecular Biosciences*.

[135] Wettstein L., Knaff P. M., Kersten C. (2022). Peptidomimetic Inhibitors of TMPRSS2 Block SARS-CoV-2 Infection in Cell Culture. *Communications Biology*.

[136] Karolyi M., Pawelka E., Omid S. (2022). Camostat Mesylate versus Lopinavir/Ritonavir in Hospitalized Patients With COVID-19—Results from a Randomized, Controlled, Open Label, Platform Trial (ACOVACT). *Frontiers in Pharmacology*.

[137] Hoffmann M., Hofmann-Winkler H., Smith J. C. (2021). Camostat Mesylate Inhibits SARS-CoV-2 Activation by TMPRSS2-Related Proteases and Its Metabolite GBPA Exerts Antiviral Activity. *EBioMedicine*.

[138] Citarella A., Dimasi A., Moi D. (2023). Recent Advances in SARS-CoV-2 Main Protease Inhibitors: From Nirmatrelvir to Future Perspectives. *Biomolecules*.

[139] Padmanabhan P., Desikan R., Dixit N. M. (2020). Targeting TMPRSS2 and Cathepsin B/L Together May Be Synergistic Against SARS-CoV-2 Infection. *PLoS Computational Biology*.

[140] Hoy S. M. (2013). Abiraterone Acetate: A Review of its Use in Patients With Metastatic Castration-Resistant Prostate Cancer. *Drugs*.

[141] Marín‐Aguilera M., Reig Ò., Milà‐Guasch M. (2019). The Influence of Treatment Sequence in the Prognostic Value of TMPRSS2‐ERG as Biomarker of Taxane Resistance in Castration‐resistant Prostate Cancer. *International Journal of Cancer*.

[142] Chanawong A., Mackenzie P. I., McKinnon R. A., Hu D. G., Meech R. (2017). Exemestane and Its Active Metabolite 17-Hydroexemestane Induce UDP-Glucuronosyltransferase (UGT) 2B17 Expression in Breast Cancer Cells. *Journal of Pharmacology and Experimental Therapeutics*.

[143] Mukherjee A. G., Wanjari U. R., Nagarajan D. (2022). Letrozole: Pharmacology, Toxicity and Potential Therapeutic Effects. *Life Sciences*.

[144] Lu Y., Liu W. (2020). Selective Estrogen Receptor Degraders (SERDs): A Promising Strategy for Estrogen Receptor Positive Endocrine-Resistant Breast Cancer. *Journal of Medicinal Chemistry*.

[145] Samuel R. M., Majd H., Richter M. N. (2020). Androgen Signaling Regulates SARS-CoV-2 Receptor Levels and Is Associated With Severe COVID-19 Symptoms in Men. *Cell Stem Cell*.

[146] Qiao Y., Wotring J. W., Zheng Y. (2023). Proxalutamide Reduces SARS-CoV-2 Infection and Associated Inflammatory Response. *Proceedings of the National Academy of Sciences of the United States of America*.

[147] Jiang H., Ouyang Q., Yin Y. (2022). Proxalutamide in Patients With AR-Positive Metastatic Breast Cancer: Results From an Open-Label Multicentre Phase Ib Study and Biomarker Analysis. *European Journal of Cancer*.

[148] Keller C., Böttcher-Friebertshäuser E., Lohoff M. (2022). TMPRSS2, a Novel Host-Directed Drug Target Against SARS-CoV-2. *Signal Transduction and Targeted Therapy*.

[149] Hu X., Shrimp J. H., Guo H. (2021). Discovery of TMPRSS2 Inhibitors From Virtual Screening as a Potential Treatment of COVID-19. *ACS Pharmacology & Translational Science*.

[150] Baby K., Maity S., Mehta C. H., Suresh A., Nayak U. Y., Nayak Y. (2021). SARS-CoV-2 Entry Inhibitors by Dual Targeting TMPRSS2 and ACE2: An In Silico Drug Repurposing Study. *European Journal of Pharmacology*.

[151] Mantzourani C., Vasilakaki S., Gerogianni V.-E., Kokotos G. (2022). The Discovery and Development of Transmembrane Serine Protease 2 (TMPRSS2) Inhibitors as Candidate Drugs for the Treatment of COVID-19. *Expert Opinion on Drug Discovery*.

[152] Abiyyi M. W., Dwira S., Bustami A., Erlina L. (2023). Therapeutic Options for COVID-19: Drug Repurposing of Serine Protease Inhibitor Against TMPRSS2. *Indonesian Journal of Medical Chemistry and Bioinformatics*.

[153] DurdaĞi S. (2020). Virtual Drug Repurposing Study Against SARS-CoV-2 TMPRSS2 Target. *Turkish Journal of Biology*.

[154] Elbadwi F. A., Khairy E. A., Alsamani F. O. (2021). Identification of Novel Transmembrane Protease Serine Type 2 Drug Candidates for COVID-19 Using Computational Studies. *Informatics in Medicine Unlocked*.

[155] Choi Y., Shin B., Kang K., Park S., Beck B. R. (2020). Target-Centered Drug Repurposing Predictions of Human Angiotensin-Converting Enzyme 2 (ACE2) and Transmembrane Protease Serine Subtype 2 (TMPRSS2) Interacting Approved Drugs for Coronavirus Disease 2019 (COVID-19) Treatment Through a Drug-Target Interaction Deep Learning Model. *Viruses*.

[156] Sharanya C. S., Wilbee D. S., Sathi S. N., Natarajan K. (2024). Computational Screening Combined With Well-Tempered Metadynamics Simulations Identifies Potential TMPRSS2 Inhibitors. *Scientific Reports*.

[157] Huggins D. J. (2020). Structural Analysis of Experimental Drugs Binding to the SARS-CoV-2 Target TMPRSS2. *Journal of Molecular Graphics and Modelling*.

[158] Oduro-Kwateng E., Soliman M. E. (2024). DON/DRP‐104 as Potent Serine Protease Inhibitors Implicated in SARS‐CoV‐2 Infection: Comparative Binding Modes With Human TMPRSS2 and Novel Therapeutic Approach. *Journal of Cellular Biochemistry*.

[159] Elmezayen A. D., Al-Obaidi A., Şahin A. T., Yelekçi K. (2021). Drug Repurposing for Coronavirus (COVID-19): In Silico Screening of Known Drugs Against Coronavirus 3CL Hydrolase and Protease Enzymes. *Journal of Biomolecular Structure and Dynamics*.

[160] Shen L. W., Mao H. J., Wu Y. L., Tanaka Y., Zhang W. (2017). TMPRSS2: A Potential Target for Treatment of Influenza Virus and Coronavirus Infections. *Biochimie*.

[161] Gunst J. D., Staerke N. B., Pahus M. H. (2021). Efficacy of the TMPRSS2 Inhibitor Camostat Mesilate in Patients Hospitalized With Covid-19-A Double-Blind Randomized Controlled Trial. *EClinicalMedicine*.

[162] Böttcher-Friebertshäuser E., Stein D. A., Klenk H. D., Garten W. (2011). Inhibition of Influenza Virus Infection in Human Airway Cell Cultures by an Antisense Peptide-Conjugated Morpholino Oligomer Targeting the Hemagglutinin-Activating Protease TMPRSS2. *Journal of Virology*.

[163] Vankadari N., Ketavarapu V., Mitnala S., Vishnubotla R., Reddy D. N., Ghosal D. (2022). Structure of Human TMPRSS2 in Complex With SARS-CoV-2 Spike Glycoprotein and Implications for Potential Therapeutics. *Journal of Physical Chemistry Letters*.

[164] Grisard H. B. d. S., Schörner M. A., Barazzetti F. H. (2024). ACE2 and TMPRSS2 Expression in Patients Before, During, and After SARS-CoV-2 Infection. *Frontiers in Cellular and Infection Microbiology*.

[165] Stopsack K. H., Mucci L. A., Antonarakis E. S., Nelson P. S., Kantoff P. W. (2020). TMPRSS2 and COVID-19: Serendipity or Opportunity for Intervention?. *Cancer Discovery*.

[166] Singh N., Decroly E., Khatib A.-M., Villoutreix B. O. (2020). Structure-Based Drug Repositioning over the Human TMPRSS2 Protease Domain: Search for Chemical Probes Able to Repress SARS-CoV-2 Spike Protein Cleavages. *European Journal of Pharmaceutical Sciences*.

[167] Gamba D., van Eijk N., Lányi K. (2024). PK/PD Investigation of Antiviral Host Matriptase/TMPRSS2 Inhibitors in Cell Models. *Scientific Reports*.

[168] Zmora P., Blazejewska P., Moldenhauer A. S. (2014). DESC1 and MSPL Activate Influenza A Viruses and Emerging Coronaviruses for Host Cell Entry. *Journal of Virology*.

